# Ontogenetic variation in the cranium of *Mixosaurus cornalianus,* with implications for the evolution of ichthyosaurian cranial development

**DOI:** 10.1186/s13358-023-00289-z

**Published:** 2023-10-05

**Authors:** Feiko Miedema, Gabriele Bindellini, Cristiano Dal Sasso, Torsten M. Scheyer, Erin E. Maxwell

**Affiliations:** 1 Staatliches Museum für Naturkunde Stuttgart, Rosenstein 1, 70191 Stuttgart, Germany; 2 Hohenheim University, Schloss Hohenheim 1A, 70599 Stuttgart, Germany; 3 Dipartimento di Scienze della Terra, Università degli Studi di Milano, Milan, Italy; 4 Dipartimento di Scienze della Terra, Sapienza Università di Roma, Rome, Italy; 5 Museo di Storia Naturale di Milano, Corso Venezia 55, 20121 Milan, Italy; 6 Universität Zürich, Paläontologisches Institut, Karl Schmid-Strasse 4, 8006 Zürich, Switzerland

**Keywords:** *Mixosaurus*, Ichthyosauria, Ontogeny, Development, Triassic, Monte San Giorgio

## Abstract

Relatively complete ontogenetic series are comparatively rare in the vertebrate fossil record. This can create biases in our understanding of morphology and evolution, since immaturity can represent a source of unrecognized intraspecific variation in both skeletal anatomy and ecology. In the extinct marine reptile clade Ichthyopterygia, ontogenetic series were widely studied only in some Jurassic genera, while the ontogeny of the oldest and most basal members of the clade is very poorly understood. Here, we investigate cranial ontogeny in *Mixosaurus cornalianus*, from the Middle Triassic Besano Formation of the Swiss and Italian Alps. This small-bodied taxon is represented by a wealth of material from multiple size classes, including fetal material. This allows us to assess ontogenetic changes in cranial morphology, and identify stages in the ontogenetic trajectory where divergence with more derived ichthyosaurs has occurred. Early ontogenetic stages of *Mixosaurus* show developmental patterns that are reminiscent of the presumed ancestral (early diverging sauropsid) condition. This is prominently visible in the late fetal stage in both the basioccipital, which shows morphology akin to basal tubera, and in the postorbital, which has a triradiate head. The ontogenetic trajectory of at least some of the cranial elements of *Mixosaurus* is therefore likely still very akin to the ancestral condition, even though the adult cranium diverges from the standard diapsid morphology.

**Supplementary Information:**

The online version contains supplementary material available at 10.1186/s13358-023-00289-z.

## Introduction

Ichthyopterygia is a clade of Mesozoic diapsid reptiles showing remarkable anatomical adaptations to a fully aquatic way of life. The limbs are modified as fins, the vertebral column becomes the primary propulsive organ, and the pelvic girdle is reduced (McGowan & Motani, 2003). In addition to these classic postcranial adaptations, the skull also becomes specialized, most notably through expansion of the antorbital region, especially the premaxilla, and changes to the neurovascular anatomy in this region (Serafini et al., 2022), specialization of the external nares (Campos et al., 2019; Massare et al., 2021), enlargement of the orbit (Motani, 2005), loss of the lower temporal bar followed by loss or closure of the lower temporal fenestra (Liu et al. 2011), exclusion of both the squamosal and postorbital from the upper temporal fenestra (Motani et al., 1998), and reduced ossification in the braincase of adults (Maisch & Matzke, 2006). The magnitude of these changes, in combination with rapid rates of morphological evolution early in the history of the clade (Moon & Stubbs, 2020), have created controversies in recognizing homologous states (e.g., the lower temporal arcade Liu et al. 2011).

Mixosauridae is a clade of small early diverging ichthyosauriforms, which inhabited the shallow seas of the Middle Triassic. Members of the clade have been found throughout the Northern Hemisphere, meaning they had a pan-Tethyan distribution as well as occurring on the eastern and western coastlines of the Panthalassa Ocean (Brinkmann, 1998; Jiang et al., 2006; Roberts et al., 2022; Schmitz et al., 2004). The group is characterized by a unique cranial morphology, comprising a mixture of primitive and derived features including high sagittal and supraorbital crests, a compact circumorbital area, long upper temporal fossae, and the retention of a lower temporal opening bordered in part by the postorbital (Liu et al., 2011; Motani, 1999). However, within the clade, both intraspecific and interspecific variation in cranial anatomy is poorly understood, leading to taxonomic instability (e.g., *Mixosaurus panxianensis* is either considered to be a basal genus sister to *Mixosaurus* + *Phalarodon* (Moon, 2017), or within *Mixosaurus* (Jiang et al., 2006)).

Ontogeny has not been extensively used as a tool with which to address questions of homology or process in the cranial evolution of early ichthyopterygians, despite available ontogenetic series including fetal material (Brinkmann, 1996; Klein et al., 2020; Miedema et al., 2023; Motani et al., 2014). Ontogenetic data were successfully applied to analogous problems in other fossil tetrapod groups [e.g., enantiornithines: (Chiappe et al., 2007), placoderms (Johanson & Trinajstic, 2014) and early archosauromorphs (Ezcurra & Butler, 2015)], and have the potential to shed new light on some classic problems in ichthyosaurian cranial evolution. Ichthyosaurian ontogeny is generally portrayed as a sequence of increasingly truncated ossification sequences [e.g., loss of perichondral ossification in the limb (Maxwell et al., 2014), loss of the basioccipital peg through delayed ossification (Miedema & Maxwell, 2019)]. However, the potential for more complex ontogenies also exists. Mixosaurids provide an excellent system in which to examine these ontogenetic changes, and many of the specializations of the skull involve increases—rather than decreases—in the amount of bone tissue present.

The first specimens of *Mixosaurus cornalianus* were discovered in the Middle Triassic Besano Formation of the southern Alpine region (Repossi, 1902). However, even though the material has been known since the late nineteenth century, the cranial osteology of *M. cornalianus* is imperfectly known, in part due to the fused sutures and complex three-dimensional morphology of the adult skulls (Brinkmann, 1998), (Motani, 1999), (Renesto et al., 2020), leading to erroneous interpretations of mixosaurid osteology. Interpretation of the material from the Besano Formation is difficult due to the flattened nature of preservation, which ensures that in no specimen the full osteology can be observed. Moreover, the 2D flattening of the material hampers a good assessment of the 3D morphology in some elements. Cranial ontogeny in the clade has never been robustly assessed, despite its potential to clarify both the origins of the crests and the exact position of sutural contacts. We addressed these issues in the type species of the genus *Mixosaurus*, *Mixosaurus cornalianus*, from the Besano Formation. This further clarifies distinct osteological traits of *Mixosaurus* and adds to our knowledge of the development of the ichthyosaur cranium. Moreover, the fetal material lends us more insight into prenatal developmental pathways of ichthyosaurs.

## Material and methods

We examined the cranial osteology of 19 well-preserved mixosaurid specimens in the collections of PIMUZ, MSNM and GPIT (Additional file 1: Table S1). Among our sample is the proposed neotype of *Mixosaurus cornalianus* PIMUZ T 2420 (Brinkmann, 1999). All specimens used can reasonably be attributed to *M. cornalianus* based on tooth morphology as compared to the proposed neotype and referred specimens of the genus *Phalarodon* and the holotype of *M. kuhnschnyderi* PIMUZ T 1324. We did not subdivide our sample in morphotypes A and B as proposed by earlier authors (Brinkmann, 1997), as most specimens did not display the features necessary for subdivision. Although mixosaurids, unlike more derived taxa, do preserve lines of arrested growth in their bone histology, they do not preserve an external fundamental system and thus histological maturity cannot be assessed using this technique (Kolb et al., 2011). The smallest postnatal individual, PIMUZ T 0077 has a mandible length of 102 mm and a humeral proximodistal length of 14 mm and was presumed to be a neonate (Kolb et al., 2011). The smallest gravid female (out of 3 known specimens), PIMUZ T 4830, has a mandible length of 208 mm and an unknown humeral proximodistal length but likely between 25 and 30 mm based on specimens of similar size. Therefore, we use a mandible length of 200 mm and above and/or humeral proximodistal length 25 mm or above as the baseline size of sexual maturity. Using mandible length as a proxy, we created three ontogenetic groups: (1) fetal—material always associated with an adult skeleton and a mandible length less than or equal to 100 mm and/or humeral length less than or equal to 15 mm; (2) juvenile—material never associated with an adult and mandible lengths 100–175 mm and/or humeral length less than 25 mm; and (3) adult (= sexually mature), mandible length 200 mm or greater and/or humeral length exceeding 25 mm. We subsequently identified osteological characters separating the two postnatal groups. Even though only one gravid female displays a humerus, the baseline for maturity on humeral size was set at 25 mm as all specimens above this humeral length showed similar cranial osteology to the known sexually mature specimens. In some descriptions in the text, the adjectives “smaller” and “larger” will be used to describe members of the juvenile stage. This is to discriminate between smaller and larger specimens of the same stage displaying objectively different morphology.

The fetal material used was determined to be similar in development to *Stenopterygius* stage 4, meaning the fetal stage observed is a relatively late (perinatal) stage of prenatal development (Miedema & Maxwell, 2022; Miedema et al., 2023).

The material studied comes from the Besano Formation, a formation belonging to the Middle Triassic carbonate succession of Monte San Giorgio, UNESCO World Heritage Site. The specimens here presented come from different localities, both from the Italian and the Swiss side of Monte San Giorgio, as summarized in Additional file 1: Table S1. The Besano Formation was deposited in a shallow marine setting (30–130 m deep) and consists of an alternation of laminated dolomitic banks and bituminous shales, with sparse cineritic tuffs (Bernasconi & Riva, 1993; Furrer, 1995; Röhl et al., 2001), which are Late Anisian–Early Ladinian in age (Brack & Rieber, 1993; Brack et al., 2005; Mundil et al., 1996; Wotzlaw et al., 2017). The material studied originates from the middle part of the Besano Formation, which is dated to the latest Anisian, was deposited in an intraplatform basin (Röhl et al., 2001), and from which a great number of ichthyosaurian remains and other pelagic vertebrates were recovered [e.g., Furrer, 2003; Bindellini et al., 2021; Brinkmann, 1997; Renesto et al., 2020; Dal Sasso & Pinna, 1996; Bindellini & Dal Sasso, 2022]. Specimens in the Besano Formation are almost ubiquitously preserved laterally or dorsoventrally flattened, however in most cases some original 3d relief is preserved even in fetal material (Miedema et al., 2020; Spiekman et al., 2020). Therefore, even though some diagenetic altering may have taken place, it should not majorly influence ontogenetic assessment and discrete ontogenetic variation observed is genuine.

## Results

### Chondrocranium

#### Basioccipital

**Fig. 1:**
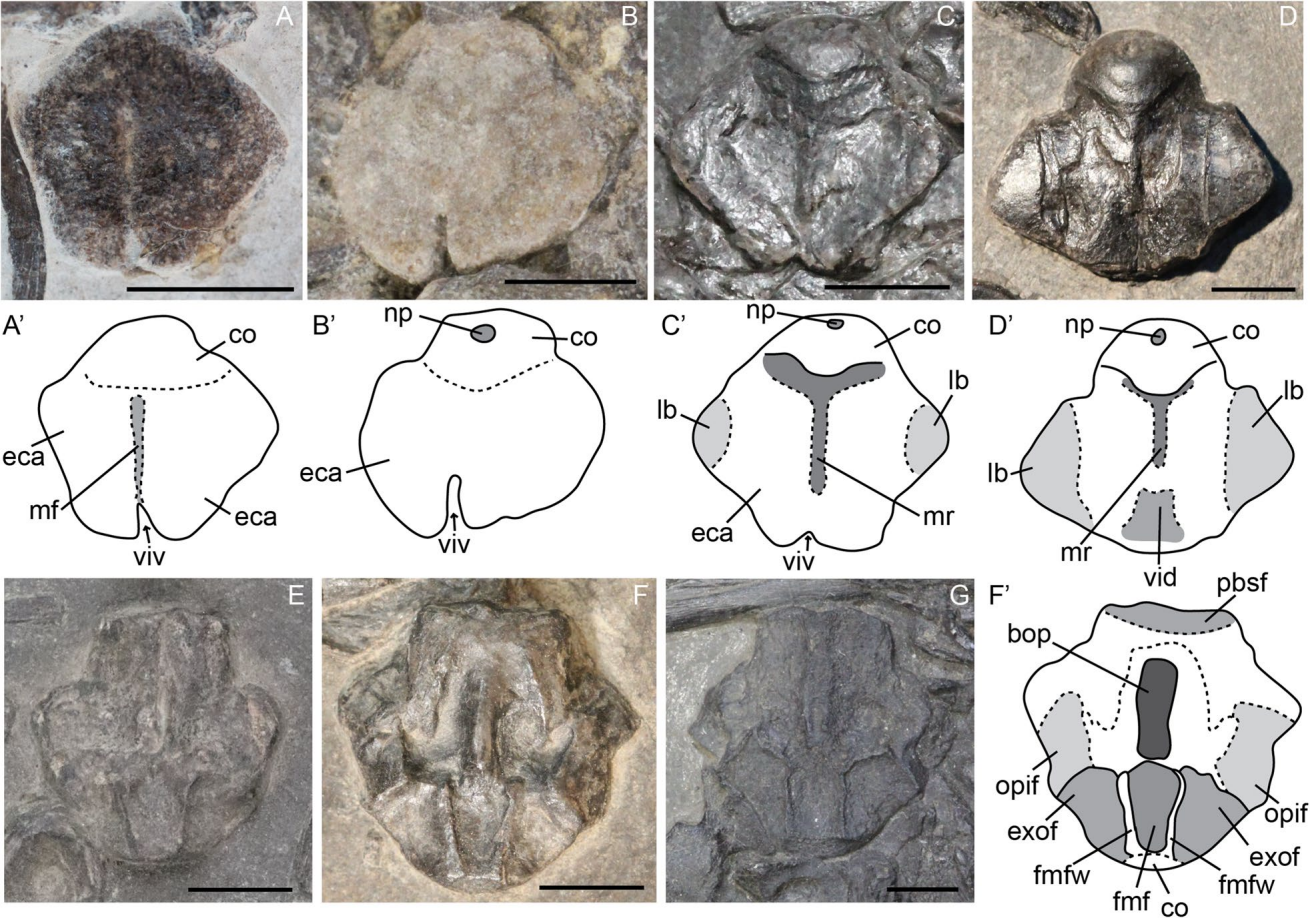
*Mixosaurus cornalianus*, basioccipital in posteroventral (**A**–**D**) and dorsal (**E**–**G**) view. Interpretative drawings are identified by an apostrophe beside their corresponding letter. **A** Fetus PIMUZ T 4830; **B** smaller juvenile PIMUZ T 0077; **C** juvenile PIMUZ T 4857; **D** adult PIMUZ T 4848; **E** juvenile PIMUZ T 2416; **F** adult PIMUZ T 2414; **G** adult MSNM BES SC 1001. *bop* basioccipital peg; *co* condyle; *eca* extracondylar area; *exof* exoccipital facet; *fmf* floor of the foramen magnum; *fmfw* foramen magnum lateral edge (wall); *lb* lateral bulge, *mf* midline furrow, *mr* midline ridge, *np* notochord pit, *opif* opisthotic facet, *pbsf* parabasisphenoid facet, *vid* ventral indentation, *viv* ventral invagination. Scale bar equals 5 mm

In posterior view the basioccipital consists of a round condyle and a wide extracondylar area. The basioccipital is still not fully ossified in the fetal stage as seen by its pitted texture. The transition between the extracondylar area and the condyle is not well developed but these regions become more delineated postnatally and are well delineated around sexual maturity (Fig. 1A–D). The notochordal pit is present in all stages. It is not visible in the only fetal specimen, but this is likely because the element is preserved in more posteroventral than directly posterior view (Fig. 1A–D). In the fetal stage, the extracondylar area has a clear ventral invagination which continues into a midline furrow and reaches to the ventral part of the condyle (Fig. 1A). This furrow and invagination are still prominently present in smaller juveniles, but do not reach the ventral margin of the condyle as in the fetal stage (Fig. 1B). Before sexual maturity, the furrow is completely gone, but a remnant of the invagination is still present; around sexual maturity, this remnant has become a ventral depression instead of an invagination (Fig. 1C, D). The midline furrow is replaced in these stages by a midline ridge, projecting ventrally from the ventral-most point of the condyle (Fig. 1C, D). The lateral wings of the extracondylar area are weakly developed in the late fetal and smaller juvenile stages (Fig. 1A, B). They develop distinct lateral bosses prior to sexual maturity. These bosses are well delineated and occupy a substantial portion of the extracondylar area around sexual maturity (Fig. 1C, D). The dorsal view of the basioccipital was only preserved in the later ontogenetic stages (juvenile to sexual maturity). In these stages the exoccipital and opisthotic facets are clearly delineated and well developed (Fig. 1E–G). The foramen magnum floor is about as wide as the exoccipital facet and has a well delineated lateral wall. Anteriorly to the foramen magnum floor is an elongated basioccipital peg approximately two-thirds of the anteroposterior length of the basioccipital in the observed postnatal stages.

#### Parabasisphenoid

**Fig. 2:**
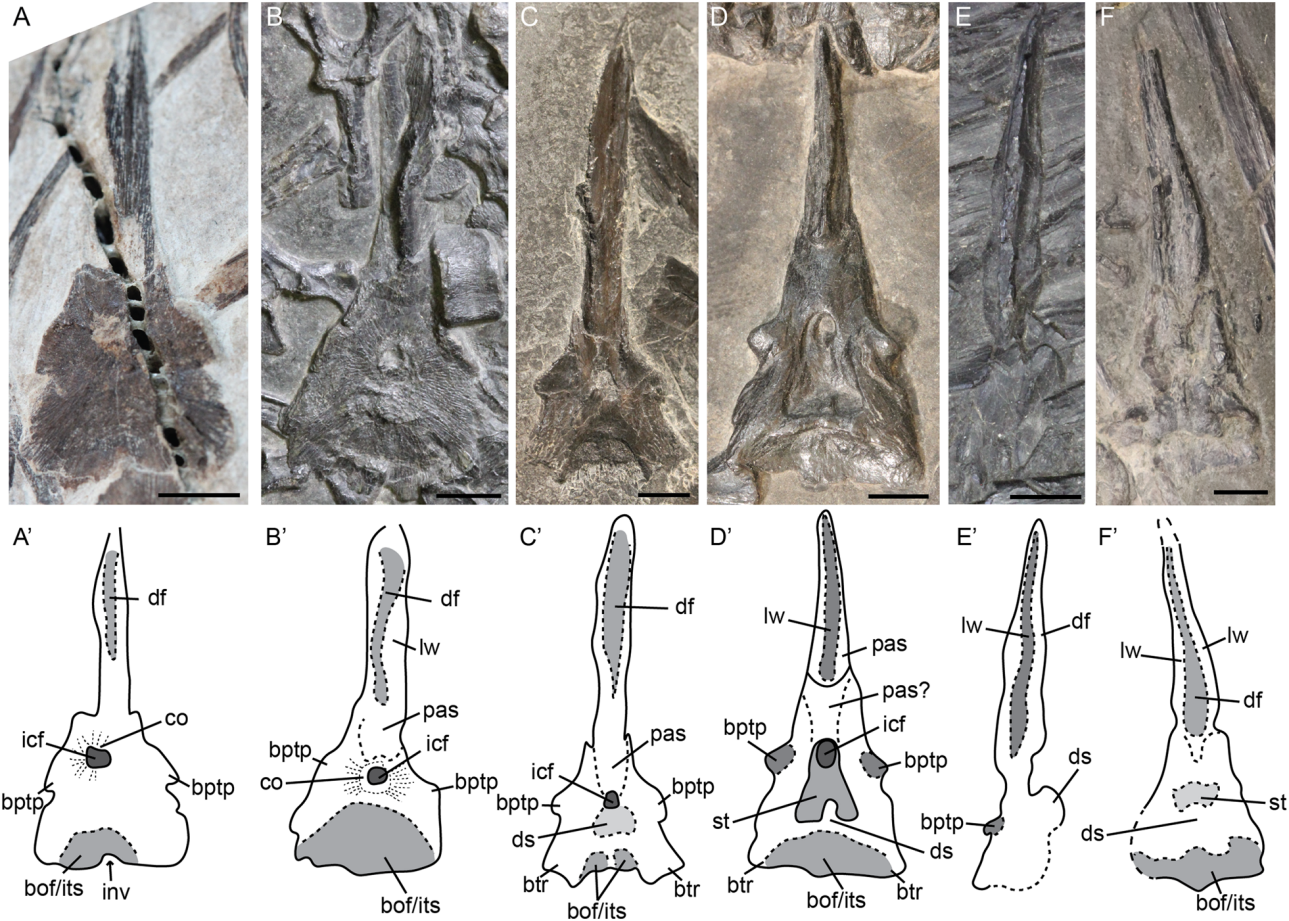
*Mixosaurus cornalianus*, parabasisphenoid in presumed dorsal (**A, C**, **D**, **F**), ventral (**B**) and left-lateral (**E**) view. Interpretative drawings are identified by an apostrophe to their corresponding letter. **A** Fetus PIMUZ T 4830; **B** larger juvenile MSNM BES SC 1903; **C** adult PIMUZ T 2407; **D** adult PIMUZ T 2414; **E** adult MSNM BES SC 1001; **F** adult GPIT PV 76273. *bof/its* basioccipital facet/intertuberal surface, *bptp* basipterygoid process, *btr* basal tuber remnant, *co* ossification centre, *df* dorsal furrow, *ds* dorsum sellae, *icf* internal carotid foramen, *inv* invagination, *lw* lateral wall, *pas* parasphenoid, *st* sella turcica, *vk* ventral keel. Scale bar equals 5 mm

We observed one fetal, one juvenile, and several adult parabasisphenoids in mostly dorsal but also ventral and lateral view (Fig. 2A–F). The parabasisphenoid in dorsoventral view is triangular in shape throughout ontogeny, whereby the width of the posterior parasphenoid is equal to the anterior width of the basisphenoid (Fig. 2B–F). Only in the fetal stage this is not fully the case (Fig. 2A). The parabasisphenoid is relatively well ossified in the fetal stage, but the ossification fibres are still visible radiating outward from the internal carotid foramen area (Fig. 2A). The fibres are still apparent postnatally (at least in ventral view) (Fig. 2B). Postnatally the parasphenoid is broad and forms a dorsally facing trough surrounded by thick lateral walls on either side and a keel ventrally (Fig. 2B–F). In the adult stage the dorsal morphology is very variable, but it is unclear to us how much of this variation is taxonomic or taphonomic. In general, the parabasisphenoid consists of two rounded structures anteriorly, which project laterally. These are interpreted as the basipterygoid processes. Posteromedial to the processes lies the sella turcica, which contains the hypophysial fossa and the dorsal exit of the internal carotid artery. This area can be very prominent (Fig. 2D) or almost completely absent (Fig. 2C) or unclear (Fig. 2F). In the cases where it is not prominently visible the dorsum sellae is more apparent (Fig. 2C, E). We do not agree with the previous interpretations of pronounced trabecular area at the posterior parasphenoid region, as the specimen this is based on is not well-preserved and this structure is not found in any other specimen (Maisch & Matzke, 1997a). Posterior on the basisphenoid the basioccipital facet is visible in dorsal view. This structure is either relatively wide or constricted by posterolaterally directed processes which we interpret as remnants of what would have developed into basal tubera in early diverging ichthyosauromorphs and other sauropsids (Fig. 2C–D). Given our observations, we distinguish two morphotypes among the studied specimens: (1) pronounced sella turcica, large basioccipital facet (2D, F) and (2) sella turcica poorly visible and basal tubera remnants present (Fig. 2C and likely E).

#### Opisthotic

**Fig. 3:**
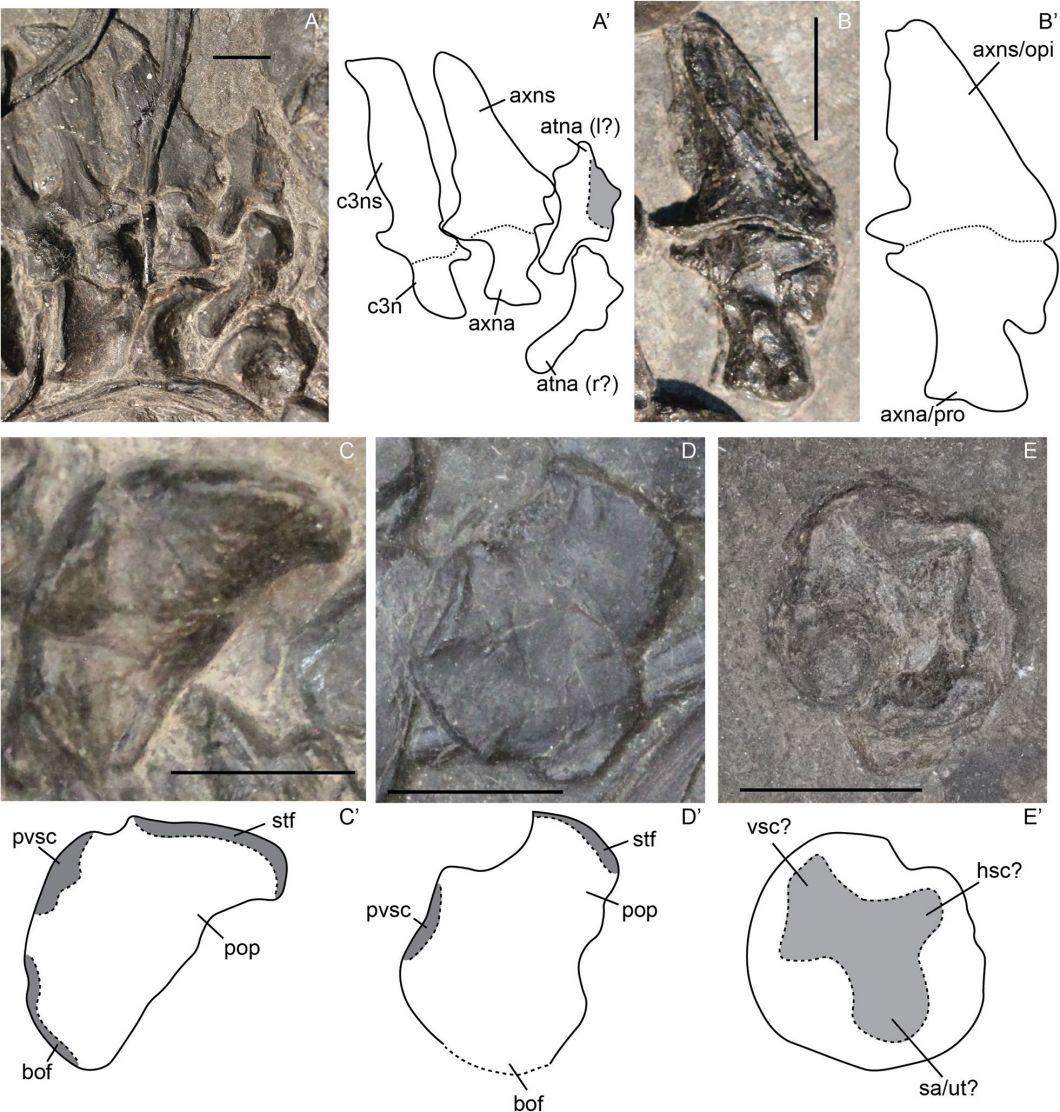
*Mixosaurus cornalianus*, anterior cervical vertebrae and otic capsule. Interpretive drawings are identified by an apostrophe alongside the corresponding letter. **A** Anterior cervical series in lateral view of adult specimen PIMUZ T 2420; **B** axis neural arch in lateral view of adult specimen PIMUZ T 4848, originally interpreted as two elements of otic capsule; **C** opisthotic in posterior view of adult specimen PIMUZ T 2414; **D** opisthotic in posterior view of adult specimen MSNM BES SC 1001. **E** prootic(?) of adult? specimen PIMUZ T 2415. *atna* atlas neural arch, *atns* atlas neural spine, *axna* axis neural arch, *axns* axis neural spine, *bof* basioccipital facet; cr3, cervical 3 neural arch; c3n, cervical 3 neural spine, *hsc* indentation for the horizontal semicircular canal, *opi* opisthotic, *pop* paroccipital process, *pro* prootic, *pvsc* process made by the indentation for the vertical semicircular canal, *sa/ut* indentation for the sacculus and/or the utriculus, *stf* supratemporal facet, *vsc* indentation for the vertical semicircular canal. Scale bar equals 5 mm

We tentatively identify an opisthotic in two adults (PIMUZ T 2414, MSNM BES SC 1001; Fig. 3C, D). The opisthotic is relatively quadrangular in outline in posterior view, with the only process protruding laterally being the paroccipital process. The areas of the basioccipital facet and the posterior vertical semicircular canal (pvsc) are about the same size and do not protrude out of the main body of the opisthotic. The area containing the pvsc is well delineated posteriorly (Fig. 3C, D). The area between the pvsc process and the paroccipital process is convex in both specimens, but more expanded in MSNM BES SC 1001. This morphological difference is attributable to non-ontogenetic intra- or inter-specific variation.

The morphology of the element we describe as the opisthotic differs greatly from that described by Maisch et al. (Maisch et al., 2006) for *Mixosaurus cornalianus*. We propose that the element described in that study (Maisch et al., 2006: Fig. 1) is a neural arch rather than the otic capsule (see “Discussion”).

#### Prootic

We tentatively identify a prootic in PIMUZ T 2416 (Fig. 3E). The prootic is visible in posterior view. It is round in outline and shows clear depressions for the semicircular canals internally. Following this interpretation, the prootic is relatively similar to that of more derived hueneosaurians (Bindellini et al., 2021; McGowan, 1973; Miedema & Maxwell, 2019; Moon & Kirton, 2016).

The morphology of the element we describe as the prootic differs greatly from that described by Maisch et al. (Maisch et al., 2006) for *Mixosaurus cornalianus*. We propose that the element described in that study is a neural arch rather than the otic capsule (see “Discussion”).

#### Exoccipital

**Fig. 4:**
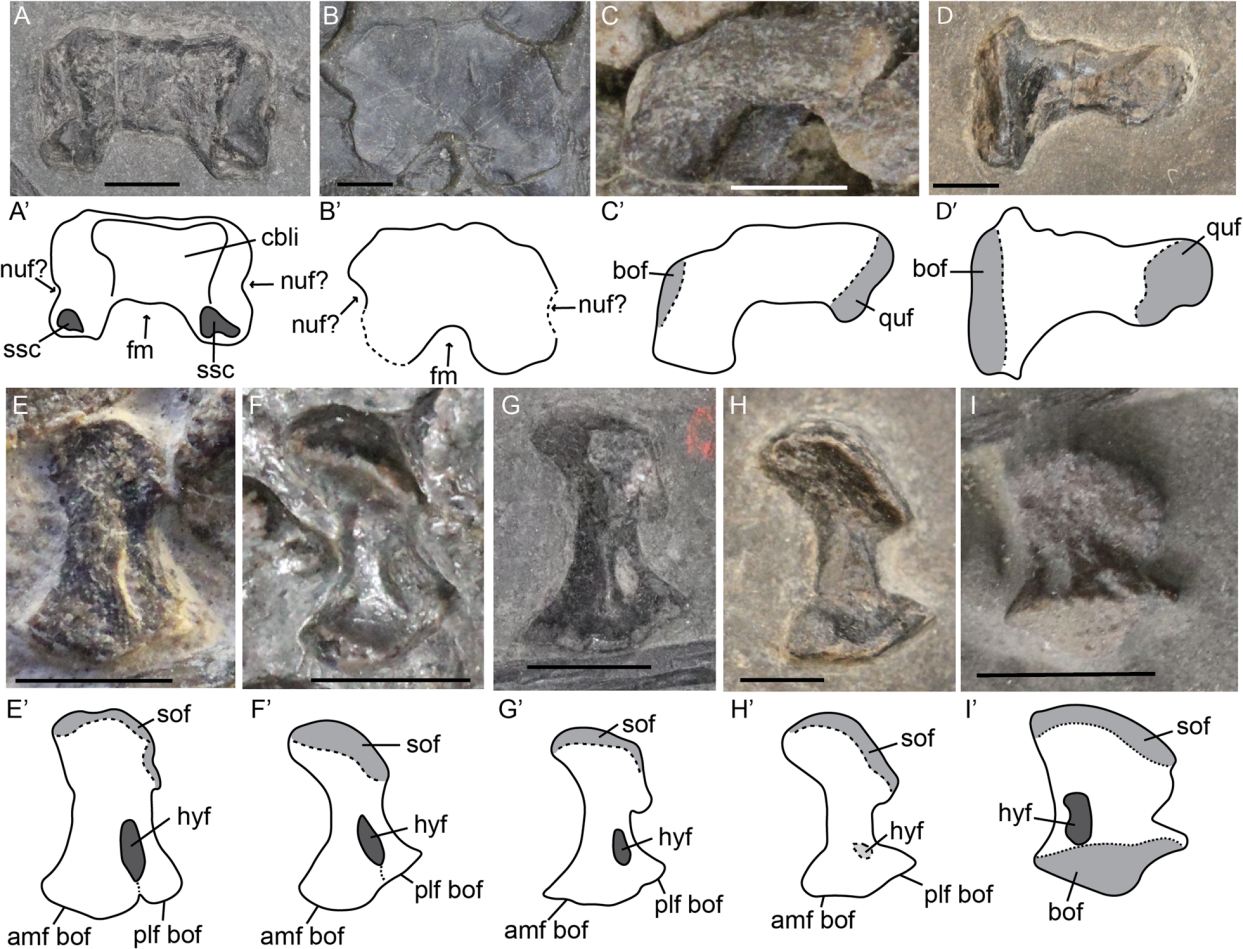
*Mixosaurus cornalianus*, supraoccipitals in anterior (**A**) and posterior (**B**) view; stapes (**C**, **D**) and exoccipitals (**E**–**H**) in posterior view and *Stenopterygius quadriscissus* exoccipital in anterior view (**I**). Interpretative drawings are identified by an apostrophe beside the corresponding letter. **A** Juvenile specimen PIMUZ T 2416; **B** adult specimen MSNM BES SC 1001; **C** juvenile specimen PIMUZ T 0077; **D** stapes in posterior view of adult specimen PIMUZ T 2414; **E** fetal specimen PIMUZ T 4830; **F** juvenile specimen PIMUZ T 4857; **G** juvenile specimen PIMUZ T 2416; **H** adult specimen PIMUZ T 2414; **I** exoccipital in anterior view of a stage 3 fetus *Stenopterygius* *quadriscissus* SMNS 80234. *amf bof* anteromedial footplate of the basioccipital facet, *bof* basioccipital facet, *cbli* indentation for the cerebellum, *fm* foramen magnum, *hyf* hypoglossal foramen, *nuf* nutritive foramen, *plf bof* posterolateral footplate of the basioccipital facet, *quf* quadrate facet, *sof* supraoccipital facet, *ssc* semicircular canal indentation. Scale bar equals 3 mm

The exoccipital displays similar levels of ossification to the parabasisphenoid and basioccipital in the fetal stage. The bone is smooth in texture in all postnatal stages. The hypoglossal foramen is about half as long as the exoccipital itself and decreases in relative size over ontogeny (Fig. 4E–H). The two footplates forming the basioccipital facets are not fully fused ventrally in the fetal stage (Fig. 4E). The suture is still visible early postnatally (Fig. 4F). This indicates that the exoccipital ossifies from a dorsal ossification centre in a ventral direction, medially and laterally surrounding the hypoglossal nerve and subsequently fusing into a footplate.

#### Supraoccipital

The supraoccipital is rarely preserved (Fig. 4A, B). We observed a supraoccipital in anterior view in a juvenile specimen and one in posterior view in a specimen around sexual maturity (likely adult). In general, the supraoccipital is approximately quadrangular in anteroposterior view, and the foramen magnum contribution is not larger than half the total height of the element. The foramen magnum contribution is wider and more quadrangular in the smaller specimen than in the larger specimen. However, whether this morphological difference is attributable to ontogeny, the view in which the element is exposed, intraspecific variation or possibly diagenetic compaction is hard to say. There are no nutritive foramina visible, but small indentations on the lateral sides of the element in both the ontogenetic stages may indicate the presence of nutritive canals close to the supraoccipital.

### Splanchnocranium

#### Stapes

Unfortunately, no definitive fetal stapes was located. In general, the stapes in sexually mature specimens is similar to other ichthyosaurs in being relatively slender, having no stapedial foramen and possessing a larger medial head (Fig. 4C, D). We do not observe the distinct kink in the stapedial shaft, nor the distinct division of the medial head as reported for *Phalarodon* cf*. fraasi* (Nicholls et al., 1999). One ontogenetic difference is observed: the adult specimen displays a dorsal projection of the medial head, this is absent in the juvenile (Fig. 4C, D). Instead, the basioccipital facet, positioned on the medial head, is incompletely ossified. This shows that the stapes is not fully ossified early postnatally.

#### Quadrate

**Fig. 5:**
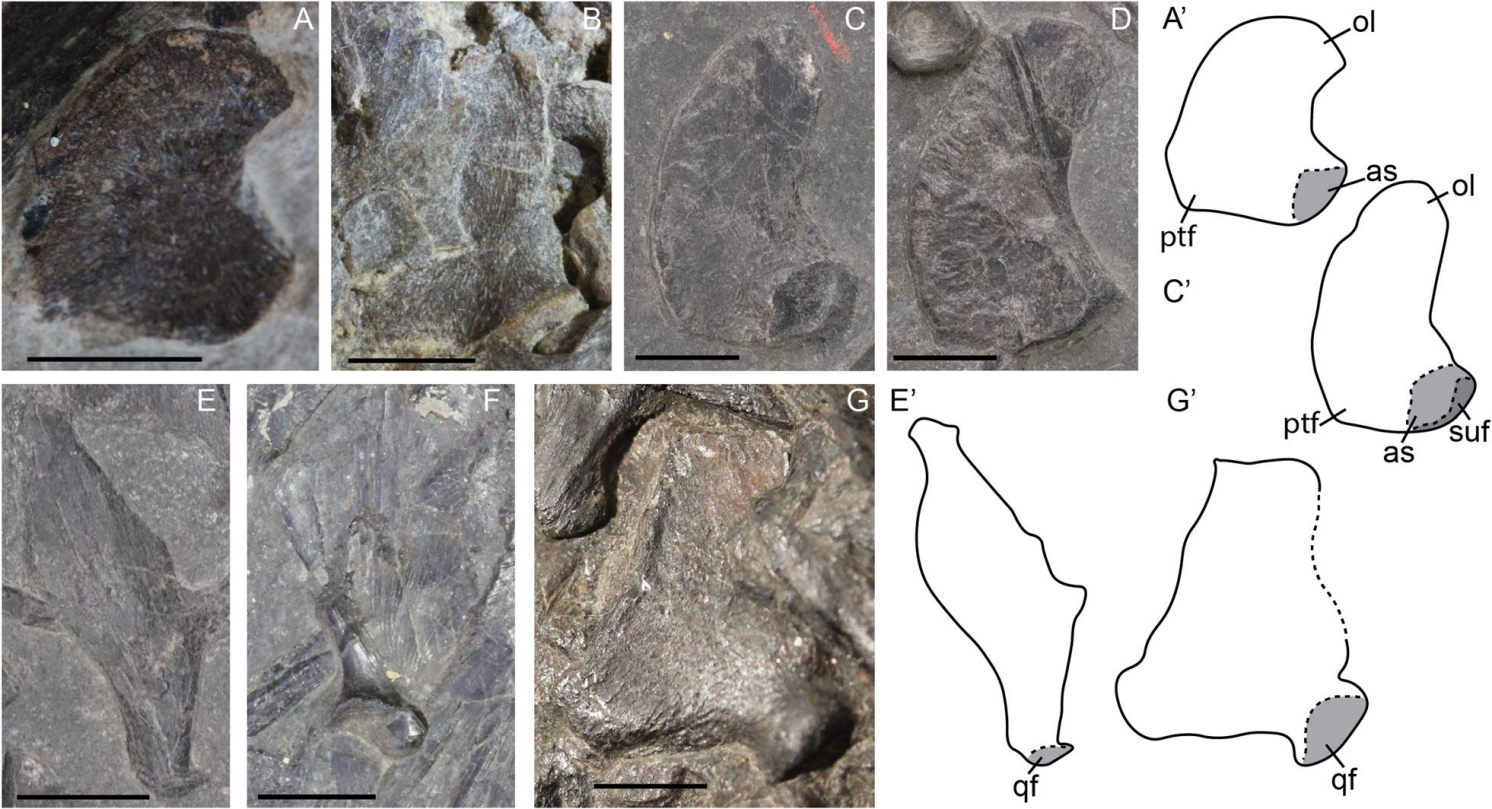
*Mixosaurus cornalianus*, quadrates and quadratojugals. Interpretative drawings are identified by an apostrophe beside the corresponding letter. **A** Isolated left quadrate of fetus PIMUZ T 4830 in lateral view; **B** semi-articulated left quadrate and quadratojugal (latter mostly hidden) of juvenile PIMUZ T 0077 in lateral view; **C** isolated left quadrate of juvenile specimen PIMUZ T 2416 in lateral view; **D** isolated right quadrate of juvenile specimen PIMUZ T 2416 in medial view; **E** isolated right quadratojugal of juvenile specimen PIMUZ T 2416 in medial view; **F** isolated right quadratojugal of a juvenile MSNM BES SC 1903 in lateral view; **G** isolated right quadratojugal of adult specimen PIMUZ T 2420 in lateral view. *as* articular surface, *ol* occipital lamella, *ptf* pterygoid flange, *qf* quadrate facet, *suf* surangular facet. Scale bar equals 5 mm

The quadrate consists of a large occipital lamella dorsally, a pterygoid flange anteroventrally and a boss containing the condyle posteroventrally. The occipital lamella is more curved perinatally and straightens dorsally over postnatal ontogeny (Fig. 5A–D). The pterygoid flange is more pronounced and protrudes relatively further from the main body in the fetal and the smaller juvenile than in the larger juvenile and adult (Fig. 5A–D), although not as much as in some shastasaur-grade ichthyosaurs in which it forms a distinct triangular process (Bindellini et al., 2021; Maisch & Matzke, 1997b).

#### Epipterygoid

No ossified epipterygoid was found across the sample. Ossified epipterygoids are found sporadically in other non-ophthalmosaurid ichthyosaurs (Bindellini et al., 2021; McGowan, 1973; Miedema & Maxwell, 2022; Yin et al., 2021), but the element is difficult to observe especially in articulated 2D-prepared specimens. It is therefore possible an ossified epipterygoid was also present in *Mixosaurus cornalianus*.

#### Articular

**Fig. 6:**
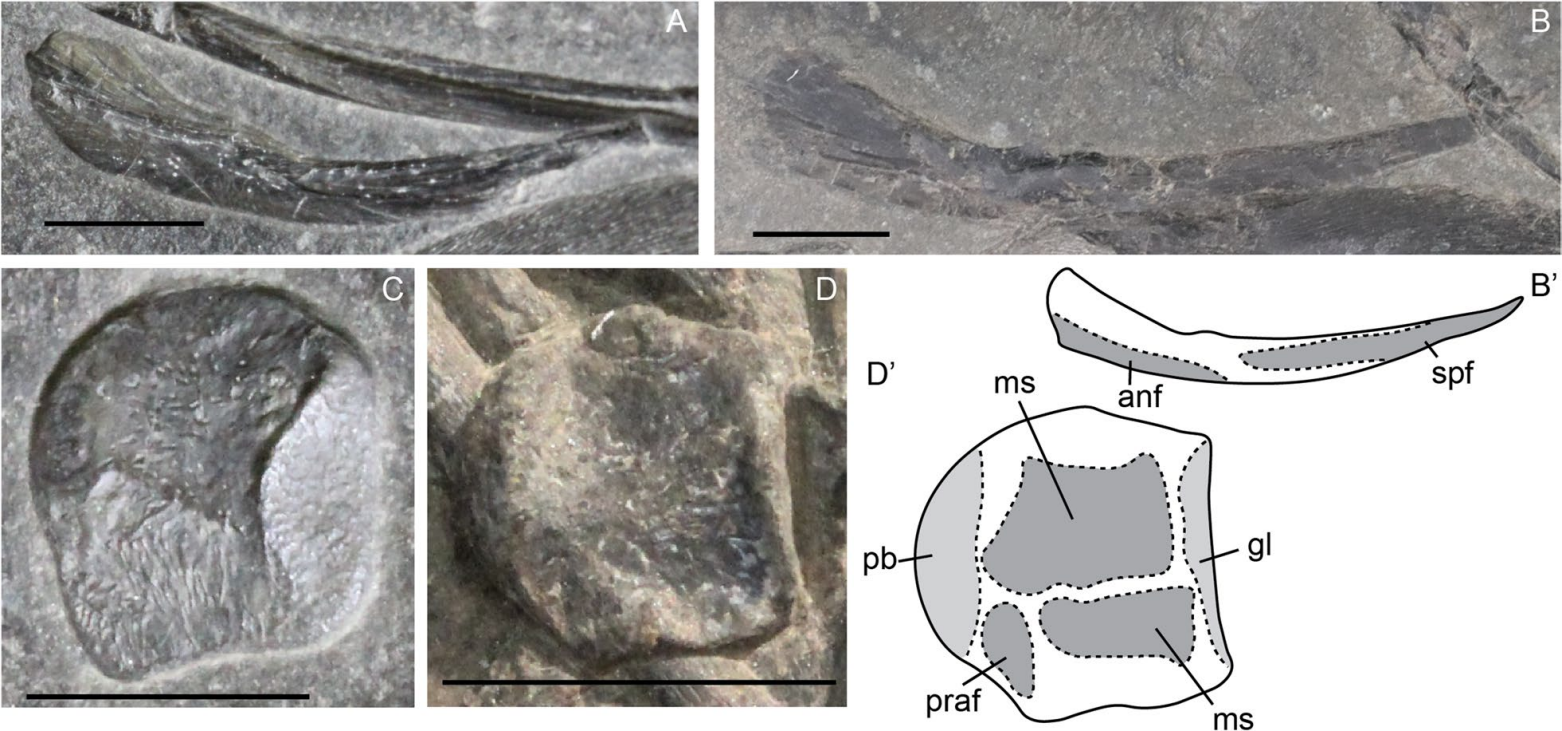
*Mixosaurus cornalianus*, isolated prearticulars and articulars. Interpretative drawings are identified by an apostrophe beside the corresponding letter. **A** Isolated left prearticular of a large juvenile MSNM BES SC 1903 in medial view; **B** isolated left prearticular of juvenile specimen PIMUZ T 2416 in medial view; **C** isolated right articular of a juvenile MSNM BES SC 1903 in medial view; **D** isolated left articular of juvenile PIMUZ T 2134 in medial view. *anf* angular facet, *gl* glenoid, *ms* medial surface, *praf* prearticular facet, *spf* splenial facet. Scale bar equals 5 mm

In our sample, we were unable to assess ontogenetic differences in the articular as we did not observe it in the fetal stage. This is possibly due to ossification level, as the articular likewise ossifies late in *Stenopterygius* (Miedema & Maxwell, 2022). Overall, the morphology is very similar to other ichthyosaurs (Fig. 6C, D). The articular is a rounded element which has a glenoid contribution over its entire anterior edge. The medial surface is divided into two major surfaces; posteroventral to these is a small facet for the prearticular.

#### Hyoid apparatus

**Fig. 7:**
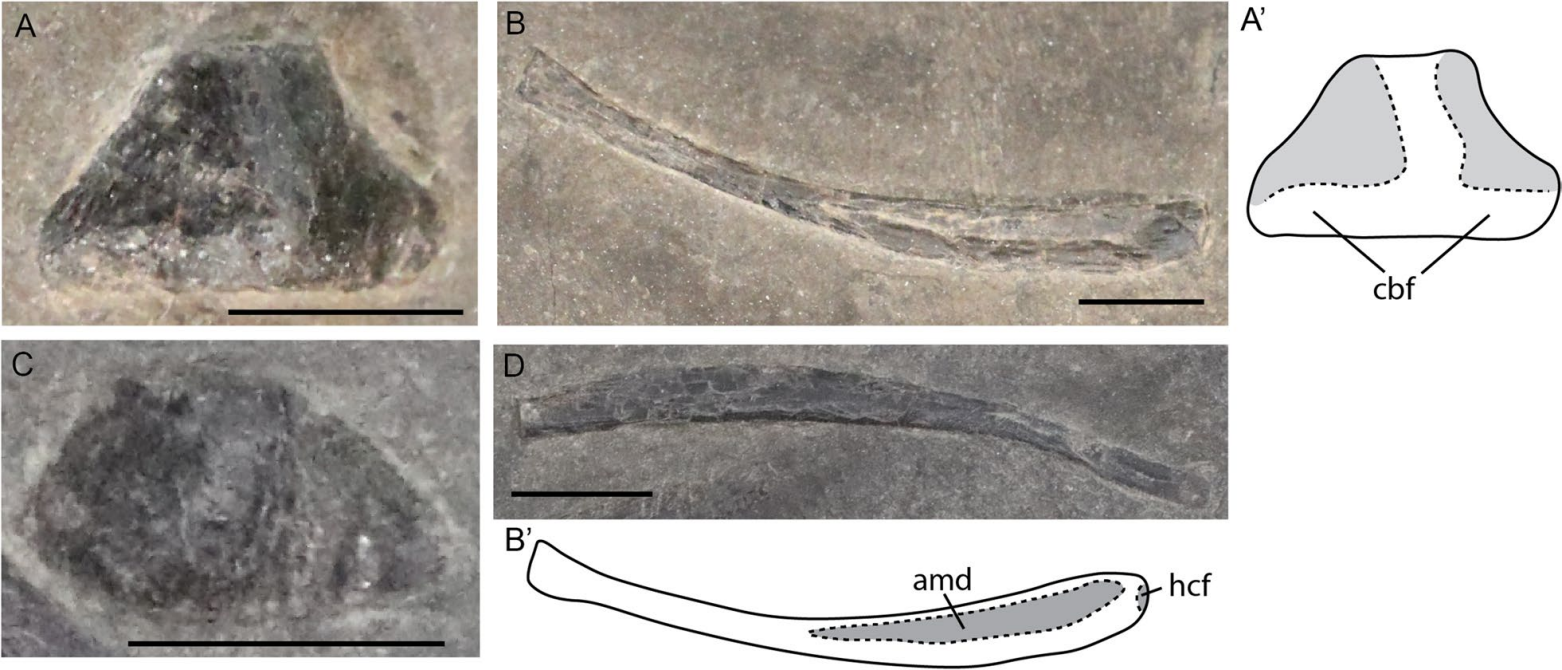
*Mixosaurus cornalianus*, isolated hyoid elements. Interpretative drawings are identified by an apostrophe beside the corresponding letter. **A** Hyoid corpus of adult specimen PIMUZ T 2414 in ventral view; **B** ceratobranchial (CB1) element of adult specimen PIMUZ T 2414 in medial? view; **C** hyoid corpus of juvenile specimen PIMUZ T 2416 in ventral view; **D** ceratobranchial (CB1) element of juvenile specimen PIMUZ T 2416 in medial? view. *amd* anteromedian depression, *cbf* ceratobranchial facet, *hcf* hyoid corpus facet. Scale bar equals 5 mm

Despite being difficult to observe, elements of the hyoid apparatus were seen in several specimens. Like all other ichthyosaurs, the apparatus in *Mixosaurus cornalianus* mainly consists of two elongated ceratobranchial elements, usually described as CB1 [note that it is not certain that these are homologous to CB1 in extant diapsids (Motani et al., 2013)] (Fig. 7B, D). In one juvenile, and one adult specimen (PIMUZ T 2414 and PIMUZ T 2416), an ossified hyoid corpus is present (Fig. 7A, C). The element is a rounded triangle in outline, similar to the morphology described in *Hauffiopteryx*, *Stenopterygius,* and *Ichthyosaurus* (Maxwell & Cortés, 2020; Miedema & Maxwell, 2022; Delsett et al., 2023). The hyoid corpus is easily missed due to its size and morphology. Moreover, the element is likely often lost and easily obscured in articulated specimens.

### Dermatocranium

Not all of the dermatocranial elements were preserved well enough in all stages to discuss their ontogeny satisfactorily. However, given that mixosaurid cranial morphology has proven challenging, we add our observations to contribute to the overall state of knowledge.

#### Premaxilla

**Fig. 8:**
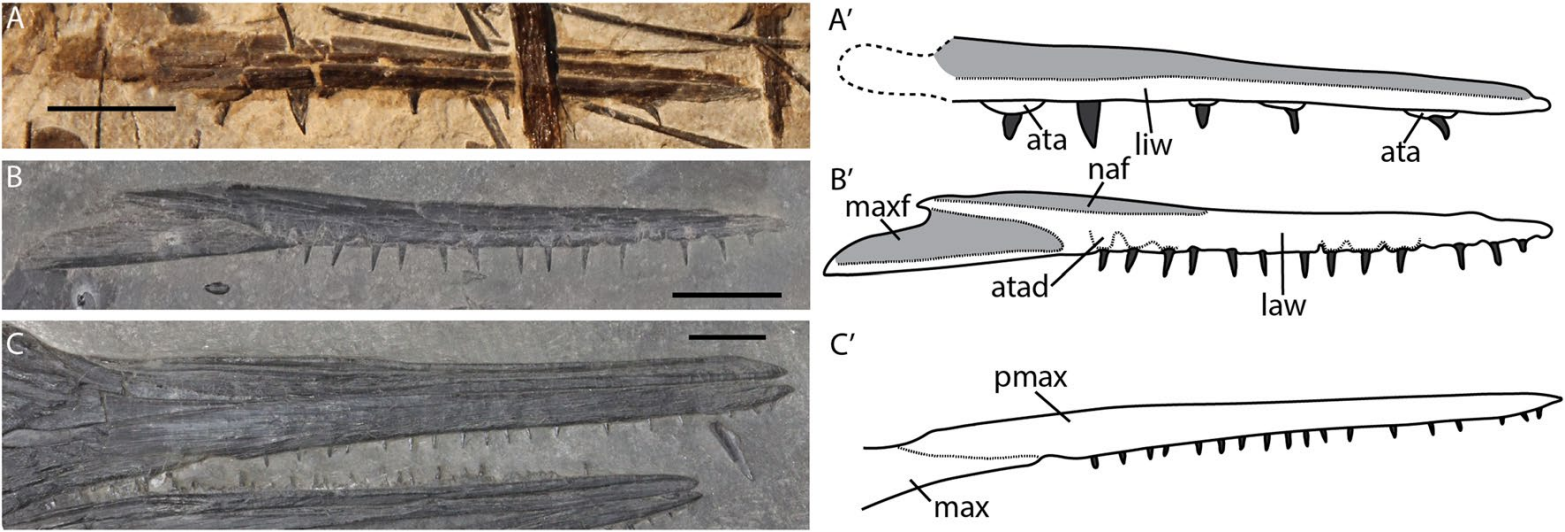
*Mixosaurus cornalianus*, isolated premaxillae. Interpretative drawings are identified by an apostrophe beside the corresponding letter. **A** Isolated premaxilla of a fetus PIMUZ T 4830 in medial view; **B** isolated premaxilla of large(?) juvenile specimen PIMUZ T 2416 in lateral view; **C** articulated rostrum of adult specimen MSNM BES SC 1001 in lateral view (mirrored image). *ata* ankylosed tooth attachment, *atad* lateral depression of ankylosed tooth attachment, *law* labial wall, *liw* lingual wall, *max* maxilla, *maxf* maxilla facet, *naf* nasal facet, *pmax* premaxilla. Scale bar equals 10 mm

The premaxilla forms the largest part of the rostrum. Seen in isolation, it bears a triangular facet for the maxilla posteriorly as well as an elongated dorsal nasal facet on its lateral surface (Fig. 8B). In isolation, the premaxilla curves upwards posteriorly, but this is almost invisible in articulation (Fig. 8B, C). The premaxilla is well ossified in the fetal stage, with no recognizable ossification centre. Tooth count increases over ontogeny, but relative tooth size decreases (Fig. 8A–C). The tooth row in ventral view was only visible in an adult specimen (GPIT PV 76274). Teeth are set in very shallow, thin-walled sockets lying in a dental groove, as previously observed (Maisch & Matzke, 1997a). The teeth are ankylosed to the premaxilla, this can be seen in lateral or medial aspect in prenatal and juvenile specimens (Fig. 8A, B).

#### Maxilla

**Fig. 9:**
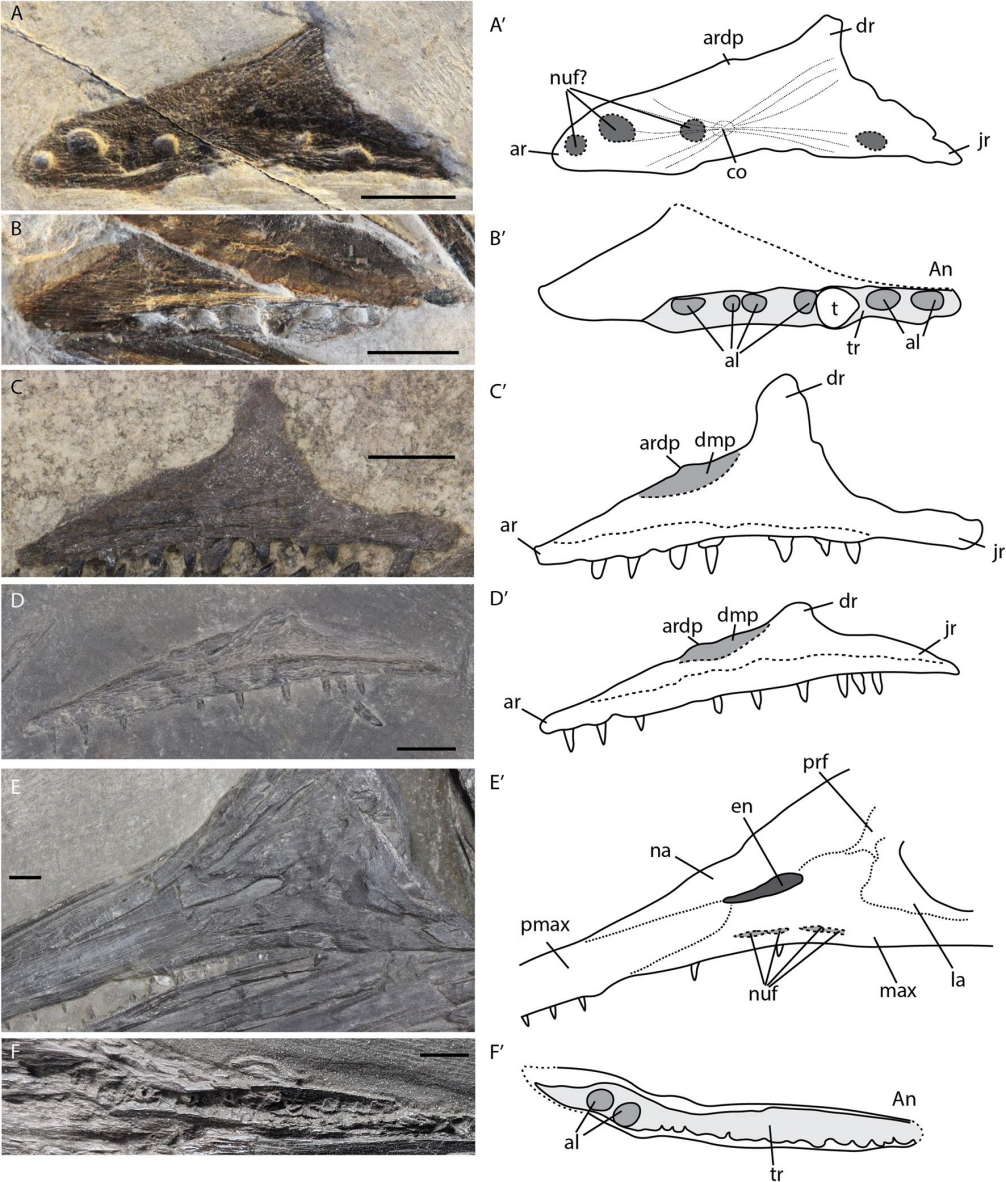
*Mixosaurus cornalianus*, maxillae. Interpretative drawings are identified by an apostrophe beside the corresponding letter. **A** Fetus PIMUZ T 4830 in medial(?) view; **B** fetus PIMUZ T 4830 in medial view; **C** juvenile specimen PIMUZ T 0077 in medial view; **D** juvenile specimen PIMUZ T 2416 in medial view; **E** adult MSNM BES SC 1001 in lateral view; **F** adult GPIT PV 76274 in ventral view. *al* alveolus, *ar* anterior ramus, *ardp* anterior ramus dorsal process, *co* ossification centre, *dmp* dorso-medial platform, *dr* dorsal ramus, *en* external naris, *jr* jugal ramus, *la* lacrimal, *max* maxilla, *na* nasal, *nuf* nutritive foramen, *pmax* premaxilla, *prf* prefrontal, *t* tooth, *tr* tooth row. Scale bar equals 5 mm

The maxilla is triangular in form in all stages (Fig. 9A–F). There is some variation in the relative size of the dorsal ramus: in one juvenile specimen it is relatively large and straight (Fig. 9C), in comparison to all other stages. We deem it likely this is at least partially due to diagenetic compression. The anterior ramus contains a small anteriorly projecting dorsal process. This process is part of the anterior-most extension of the external naris. The process is present throughout all stages. The teeth are set in distinct sockets in the fetal stage over the entire length (Fig. 9B). However, postnatally the dividing walls of the anterior sockets are thinner or even absent (Fig. 9B, F). The teeth are likely ankylosed to the tooth row or socket throughout ontogeny over the entire length of the maxilla as can be seen by the impressions laterally in some specimens (Fig. 9D). The number of tooth positions is difficult to discern, being 6 or 7 in the two fetal maxillae and at least 9 postnatally. It is therefore possible *Mixosaurus* acquired more tooth positions over postnatal ontogeny.

#### Postorbital

**Fig. 10:**
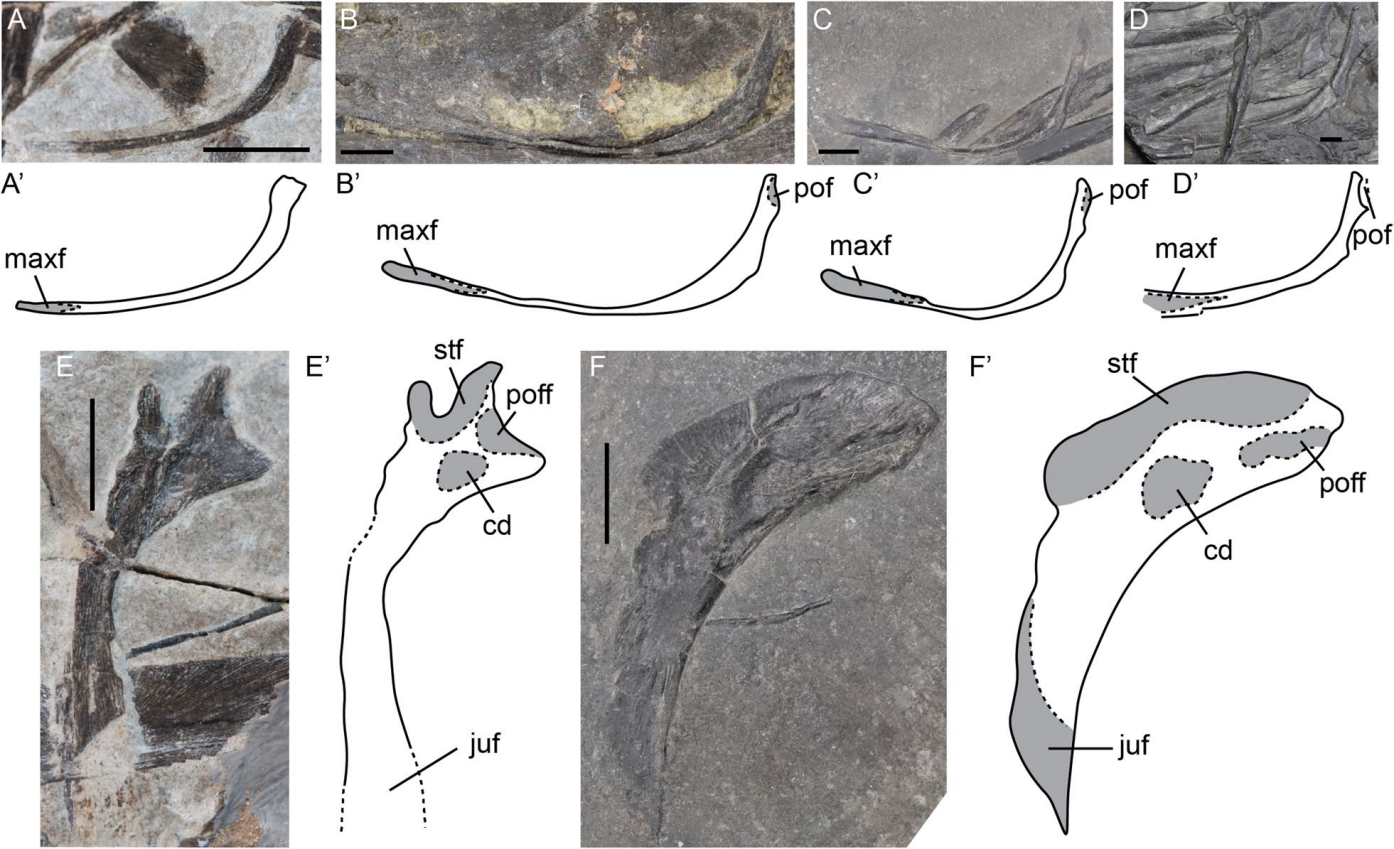
*Mixosaurus cornalianus*, isolated and disarticulated jugals and postorbitals. Interpretative drawings are identified by an apostrophe beside the corresponding letter. **A** Isolated jugal of fetus PIMUZ T 4830 in lateral? view; **B** disarticulated jugal of juvenile PIMUZ T 0077 in lateral view; **C** disarticulated jugal of juvenile specimen PIMUZ T 2416 in lateral? view; **D** semi-articulated jugal of adult MSNM BES SC 1001 in lateral view; **E** isolated postorbital of fetal specimen PIMUZ T 4830 in lateral view; **F** isolated postorbital of juvenile specimen PIMUZ T 2416 in lateral view. *cd* central depression, *juf* jugal facet, *maxf* maxilla facet, *pof* postorbital facet, *poff* postfrontal facet, *stf* supratemporal facet. Scale bar equals 5 mm

The postorbital forms the posterior margin of the orbit. It is overlapped by the supratemporal dorsally, the postfrontal anterodorsally, and the jugal ventrally. The anterior margin is concave to form the posterior rim of the orbit. The postorbital contains a central depression in lateral view. Disarticulated postorbitals in the late fetal and the large juvenile stages were identified. The anterior (orbital) curvature and central depression are similar in both cases. However, the morphology of the dorsal facets is markedly different. The fetal postorbital shows three distinct processes which include two areas of facets: the posterior one includes the supratemporal facet and the anterior one the postfrontal facet. In contrast, the juvenile specimen does not have these distinct processes, but instead the supratemporal and postfrontal facets are connected by a continuous sheet of bone (Fig. 10E, F).

#### Jugal

The jugal forms most of the ventral margin of the orbit. In the fetal stage, it is lunate in outline (Fig. 10A). Postnatally, a posterior bulge appears and the postorbital facet is better-ossified (Fig. 10A). In larger juveniles and in adults, the angle between the anterior maxillary ramus and the dorsal postorbital ramus is more acute, almost to 90 degrees, whereas the angle is more obtuse early in ontogeny (Fig. 10B–D) (the element in Fig. 10D is slightly affected by compaction).

#### Lacrimal

**Fig. 11:**
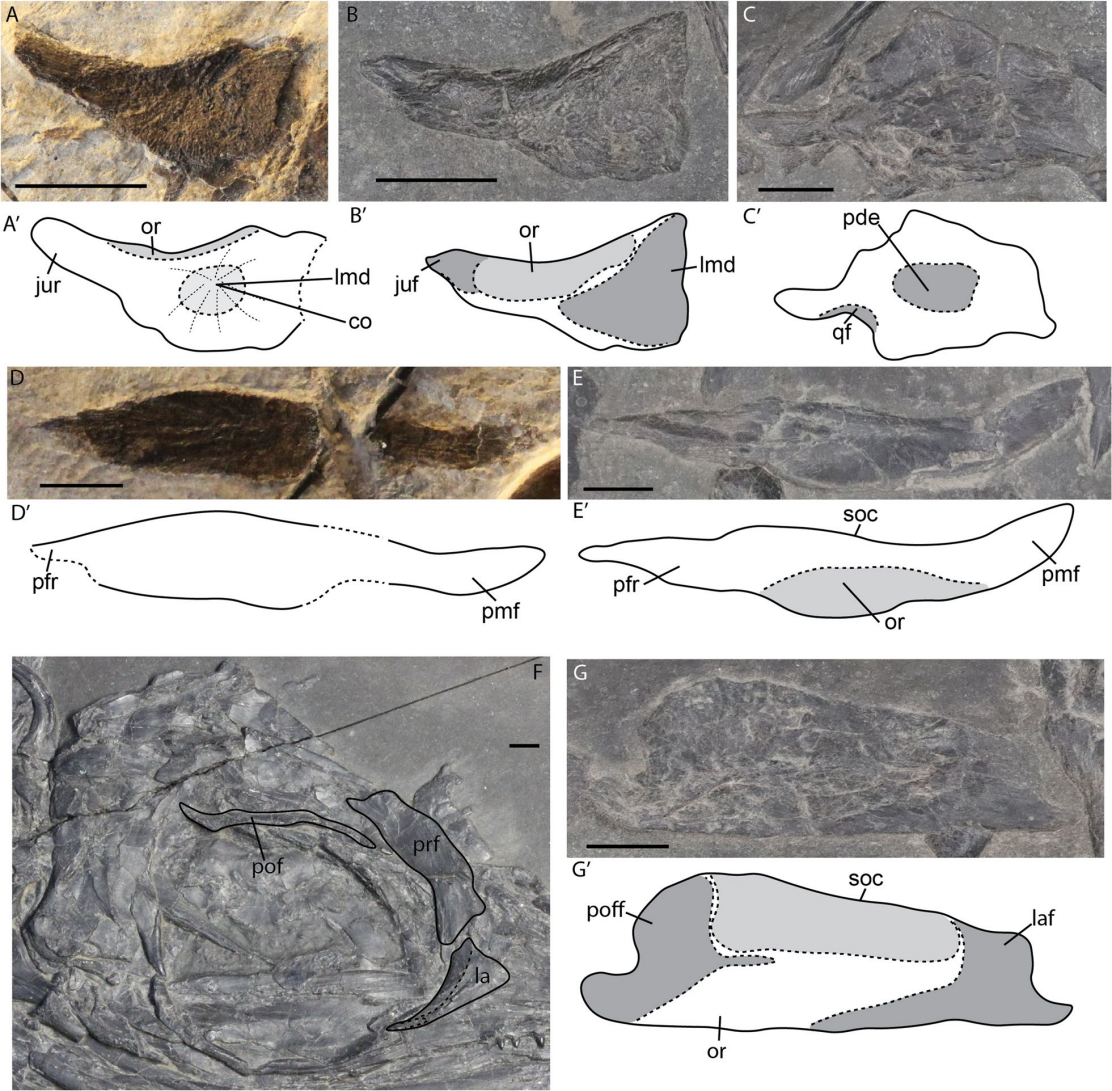
*Mixosaurus cornalianus*, isolated circumorbital and skull roof elements. Interpretative drawings are identified by an apostrophe beside the corresponding letter. **A** Lacrimal of fetus PIMUZ T 4830 in medial view; **B** lacrimal of juvenile specimen PIMUZ T 2416 in medial view; **C** supratemporal of juvenile specimen PIMUZ T 2416 in posterior view; **D** postfrontal of fetus PIMUZ T 4830 in dorsal? view; **E** postfrontal of juvenile specimen PIMUZ T 2416 in dorsal view; **F** circumorbital area of adult MSNM BES SC 1000 in lateral view; **G** prefrontal of juvenile specimen PIMUZ T 2416 in dorsal view. *co* ossification centre, *juf* jugal facet, *jur* jugal ramus, *la* lacrimal, *laf* lacrimal facet, *lmd* lacrimal medial depression, *or* orbital roof/orbital rim, *pde* posterodorsal edge supratemporal, *pfr* prefrontal ramus, *pmf* posteromedial flange, *pof* postfrontal, *poff* postfrontal facet, *prf* prefrontal, *qf* quadrate facet, *soc* supraorbital crest. Scale bar equals 5 mm

The lacrimal is a triangular element forming the anteroventral margin of the orbit. Postnatally, it has a prominent dorsal ridge, which forms the orbital margin; this ridge is not apparent perinatally (Fig. 11A, B). Medially, there is a distinct depression, which could contain the salt gland (Wahl, 2012) (Fig. 11A, B). The ossification centre is placed directly over this depression (Fig. 11A).

#### Squamosal

**Fig. 12:**
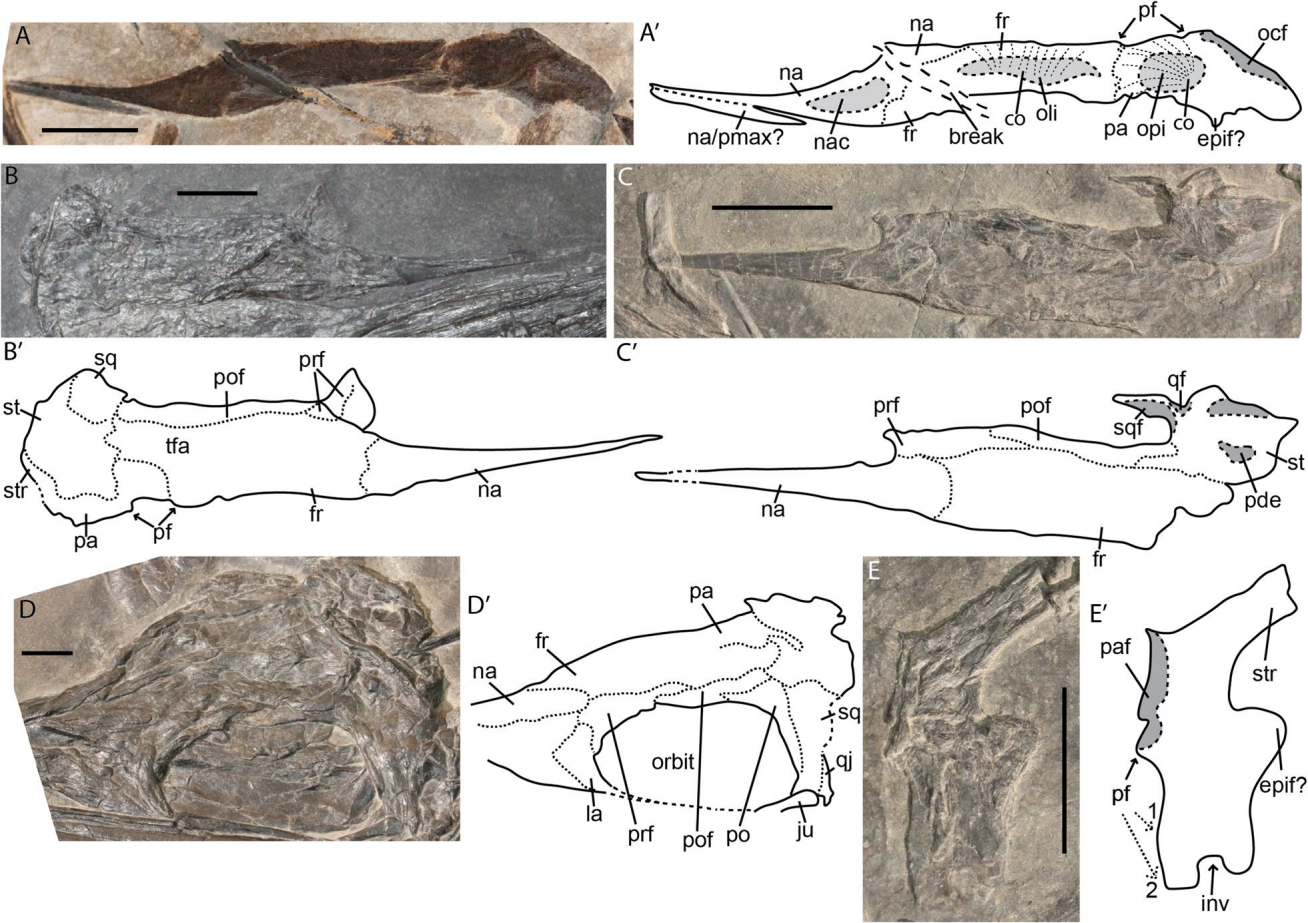
*Mixosaurus cornalianus*, skull roof elements. Interpretative drawings are identified by an apostrophe beside their corresponding letter. **A** Right-skull roof complex of fetus PIMUZ T 4830 in ventral (internal) view; **B** left-skull roof complex of juvenile PIMUZ T 4857 in dorsal view; **C** partial skull roof complex of juvenile PIMUZ T 2134; **D** skull roof of adult specimen PIMUZ T 2414 in dorsolateral view; **E** isolated parietal of small? juvenile PIMUZ T 2134 in dorsal view, numbers denote possible anterior margins of the parietal foramen. *co* ossification centre, *epif* epipterygoid facet, *fr* frontal, *inv* invagination, *la* lacrimal, *na* nasal, *nac* nasal capsule, *ocf* occipital flange, *oli* indentation for the olfactory lobe, *opi* indentation for the optic lobe, *pa* parietal, *paf* parietal facet, *pde* posterodorsal edge supratemporal, *pf* parietal foramen, *pmax* premaxilla, *prf* prefrontal, *po* postorbital, *pof* postfrontal, *qj* quadratojugal, *qf* quadrate facet, *sq* squamosal, *sqf* squamosal facet, *st* supratemporal, *str* supratemporal ramus, *tfa* anterior terrace upper temporal fenestra. Scale bar equals 10 mm

We did not manage to identify the squamosal as an isolated element. It is difficult to assess to exact morphology in articulated slab mount specimens. The squamosal is fused to the rest of the cheek area in adult specimens and (if our interpretation is correct) is a relative quadrangular dorsoventrally elongated element (Fig. 12D).

#### Quadratojugal

The quadratojugal forms the medial side of the cheek area, and is laterally overlapped by the squamosal and postorbital. In slab mount specimens it is therefore often obscured in lateral view (Fig. 12D). We observed a few isolated quadratojugals in postnatal specimens. The quadratojugal consists of a posteroventrally directed boss containing the quadrate facet, and a triangular flat dorsal portion. There is a possible ontogenetic difference between smaller juveniles and adults. In the latter, the dorsal flange is much wider and more triangular than in the immature specimens (Fig. 5E–G). However, given the low number of quadratojugals and the seemingly major postnatal ontogenetic change, we cannot say with confidence whether this is a possible ontogenetic, taxonomic, or taphonomical difference.

#### Postfrontal

The postfrontal is an elongated element forming most of the dorsal postorbital roof (Figs. 11E, 12C, D). The postfrontal tapers anteriorly at its prefrontal ramus. Posteriorly it has a posteromedial flange. There are no apparent ontogenetic shape changes of the postfrontal (Fig. 11D–F). However, the orbital roof is more prominently delineated postnatally. Both on isolated elements and on 2D-prepared specimens the supraorbital crest sensu (Maisch & Matzke, 2000) is not well detectable. The overall morphology of the postfrontal is similar to *Phalarodon atavus* and *Phalarodon fraasi* (Motani, 1999; Roberts et al., 2022).

#### Prefrontal

Like the postfrontal, the prefrontal is a difficult element to study as it is mostly found crushed in tight contact with the rest of the circumorbital bones or skull roof. We identified two disarticulated prefrontals in a juvenile specimen and in an adult (Fig. 11F, G). The prefrontal is an elongated quadrangular element that forms the anterodorsal margin of the orbit. It is overlapped by the postfrontal posterodorsally, and by the lacrimal anteriorly as can be seen by its facets (Fig. 11G). Laterally, it has a large surface which covers part of the orbit. Like in the postfrontal, the contribution to the supraorbital crest is not clear, but likely present.

#### Skull roof (parietal, frontal, nasal, supratemporal)

The skull roof of mixosaurids is often poorly preserved because of its in vivo topography and the type of preservation. We observed disarticulated skull roofs in the late fetal and small postnatal stages. In the fetal stage, the parietal, frontal and nasal are already well connected, but suture lines are still visible (Fig. 12A). These suture lines are virtually obliterated postnatally (Fig. 12B–D). The fetal skull roof observed in ventral view shows indentations for the optic lobe on the parietal, the olfactory lobe on the frontal and the nasal capsule on the posterior nasal, as in euichthyosaurians (Marek et al., 2015; McGowan, 1973; Miedema & Maxwell, 2022) (Fig. 12A). The upper temporal fenestra (UTF) is rarely visible in slab specimens due to compression of the parietal and supratemporal (Fig. 12A–D).

The nasal is a slender, elongated element. Dorsally it forms a round scarf or step joint with the frontal and contacts the prefrontal (Fig. 12A–D). The material did not allow for the identification of ontogenetic changes in our sample.

The frontal is the largest element in the skull roof. It is a large sheet, rectangular in outline. It is excluded from the orbital roof but forms most of the anterior terrace of the upper temporal fenestra (but is excluded from the anterior margin of the UTF by the parietal), which agrees with previous observations in mixosaurids (Motani, 1999). In the late fetal stage, the frontal does not extend laterally to form the lamina of bone covering the anterior terrace of the upper temporal fenestra, but rather is restricted to a medial position (Fig. 12A).

We have observed an isolated flattened parietal in a juvenile (Fig. 12E). The parietal is a complex element. Its quadrangular anterior portion is divided into the sagittal crest and the floor of the temporal fossa, separated by an anterior invagination. A large medial facet for the antimeric parietal and a posterolaterally projecting supratemporal ramus are present. We tentatively identify an epipterygoid facet laterally on the parietal. The facet would have been projected more ventrally in vivo. The parietal is the largest contributor to the parietal foramen as it either fully encloses the parietal foramen or is excluded solely from the anterior tip by the frontal (Fig. 12E). There are substantial differences between the fetal and postnatal morphology (Fig. 12A, E). The supratemporal ramus is much broader in the fetal stage than postnatally. Moreover, the antimeric facet is not yet established prenatally and the parietal foramen is small relative to the postnatal stages (Fig. 12A, E). In addition, the parietal does not contribute to the anterior edge of the upper temporal fenestra in fetuses, but this anterior lateral process has increased in size substantially by the juvenile stage (Fig. 12A, E). Three flattened supratemporals were observed in juvenile specimens (Figs. 11C, 12B, C). The supratemporal is generally round in outline, whereby its processes do not project outward substantially. The posterodorsal edge is slightly thickened and is thereby elevated on flattened specimens.

#### Palatal elements

**Fig. 13:**
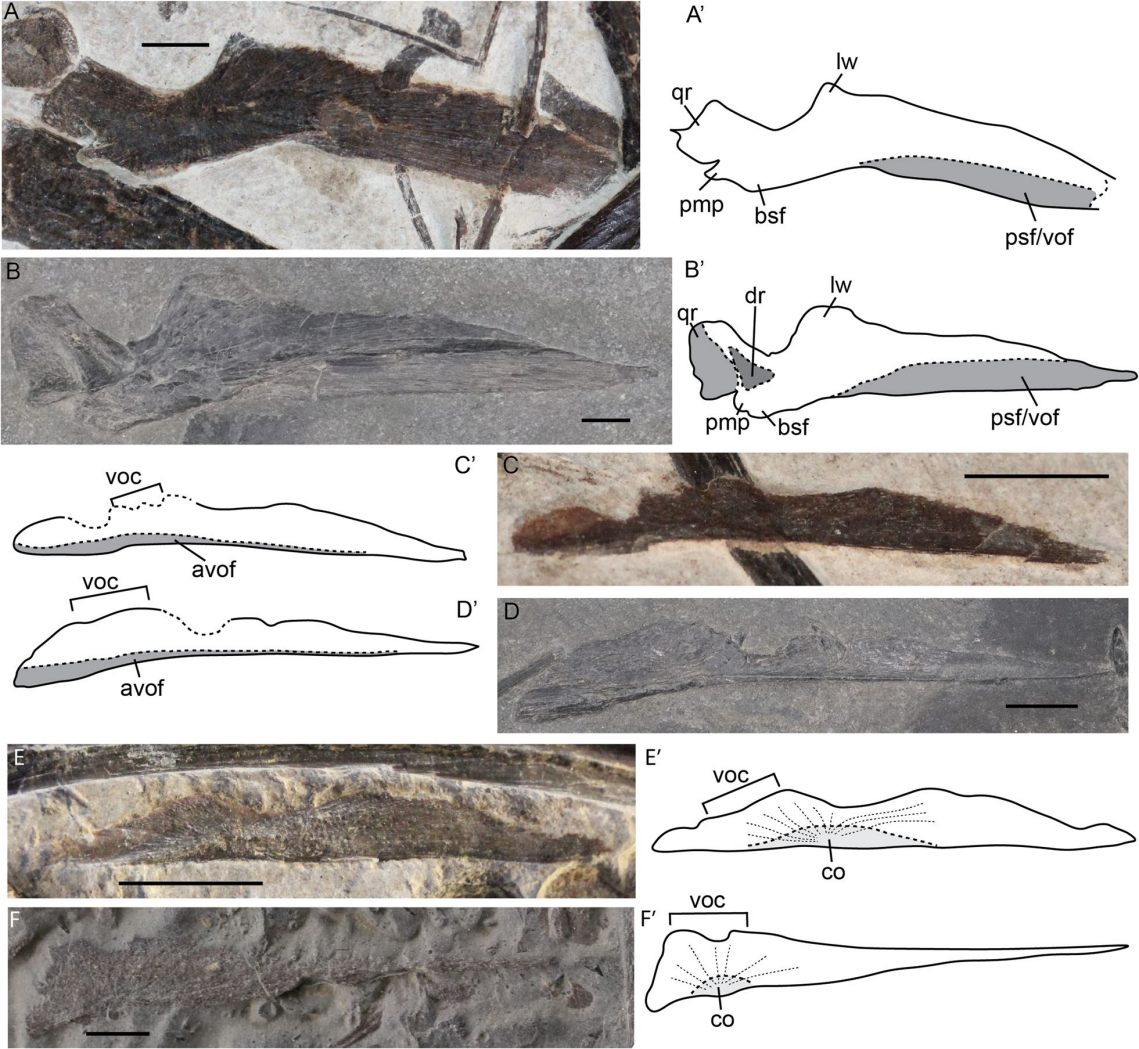
*Mixosaurus cornalianus* (**A**-**E**) and *Stenopterygius quadriscissus* (**F**) palatal elements. Interpretative drawings are identified by an apostrophe beside the corresponding letter. **A** Pterygoid of fetus PIMUZ T 4830 in dorsal view; **B** pterygoid of juvenile specimen PIMUZ T 2416 in dorsal view; **C** vomer of fetus PIMUZ T 4830 in medial view; **D** vomer of juvenile specimen PIMUZ T 2416 in medial(?) view; **E** vomer of fetus PIMUZ T 4830 in medial view; **F** vomer of stage 3 fetus SMNS 80234 in medial view. *avof* antimeric vomer facet, *bsf* basisphenoid facet; *co*, ossification centre, *dr.* dorsal ramus, *lw* lateral wing, *pmp* posteromedial process, *psf/vof* parasphenoid and vomer facet, *qr* quadrate ramus, *voc* vomer crest. Scale bar equals 5 mm

We observed isolated pterygoids and vomers in the fetal stage and in one juvenile specimen. The pterygoid consists mainly of a large, anteriorly tapering, triangular sheet-like palatal ramus and a more quadrangular quadrate ramus, separated by a parabolic medial constriction behind the lateral wing forming the subtemporal fenestra. The quadrate ramus contains a quadrate flange laterally and a relatively small dorsal flange, both of which are ossified prenatally. The medial constriction forming the medial wall of the subtemporal fossa is much wider perinatally than postnatally (Fig. 13A, B). The lateral wing is more prominent postnatally (Fig. 13A, B). The quadrate ramus is projected more laterally postnatally than perinatally (Fig. 13A, B). We deem this morphology not impacted by potential compaction. Both ontogenetic stages display a short posteromedial process (sensu Maisch & Matzke, 1997a), and its position is strongly suggestive that it is homologous with the medial flange of euichthyosaurians. Our observations regarding its morphology are not consistent with those of Maisch & Matzke, 1997a in terms of relative size. The specimen on which this was based, GPIT PV 76275 (formerly GPIT 1799/2), does not preserve the pterygoid well enough to assess its morphology.

We did not observe a clear palatine in our sample. After re-examining GPIT PV 76274 (formerly GPIT 1799/1), we tentatively agree with the interpretation of Maisch & Matzke, 1997a, although the specimen is heavily crushed and difficult to assess.

The vomer forms the anterior portion of the palate. We observed two fetal and one postnatal vomer in medial view (Fig. 13C–E). The vomer is an elongated element and tapers anteriorly. It has two distinct bulges dorsally, the posterior of which could be homologous to the vomerine crest sensu (Moon & Kirton, 2016). The ossification centre in the fetal vomer is situated anterior to the vomerine crest (Fig. 13E). Postnatally the medial antimeric vomerine facet is more pronounced than perinatally (Fig. 13C–E).

#### Dermatocranial lower jaw

The dermatocranial portion of the lower jaw consists of the surangular posteriorly, the angular posteroventrally, the prearticular posteromedially, the splenial medially, and the dentary anteriorly.

The prearticular was only observed in juvenile specimens (Fig. 6A, B). It is an elongated element, which is widest posteriorly, tapers anteriorly, and deepens in the midsection. It has elongated facets for the angular ventrally and the splenial anteriorly on its lateral surface. This morphology is consistent with many other ichthyosaurs (Kear, 2005; Marek et al., 2015; McGowan, 1973; Miedema & Maxwell, 2022; Zverkov et al., 2021).

**Fig. 14:**
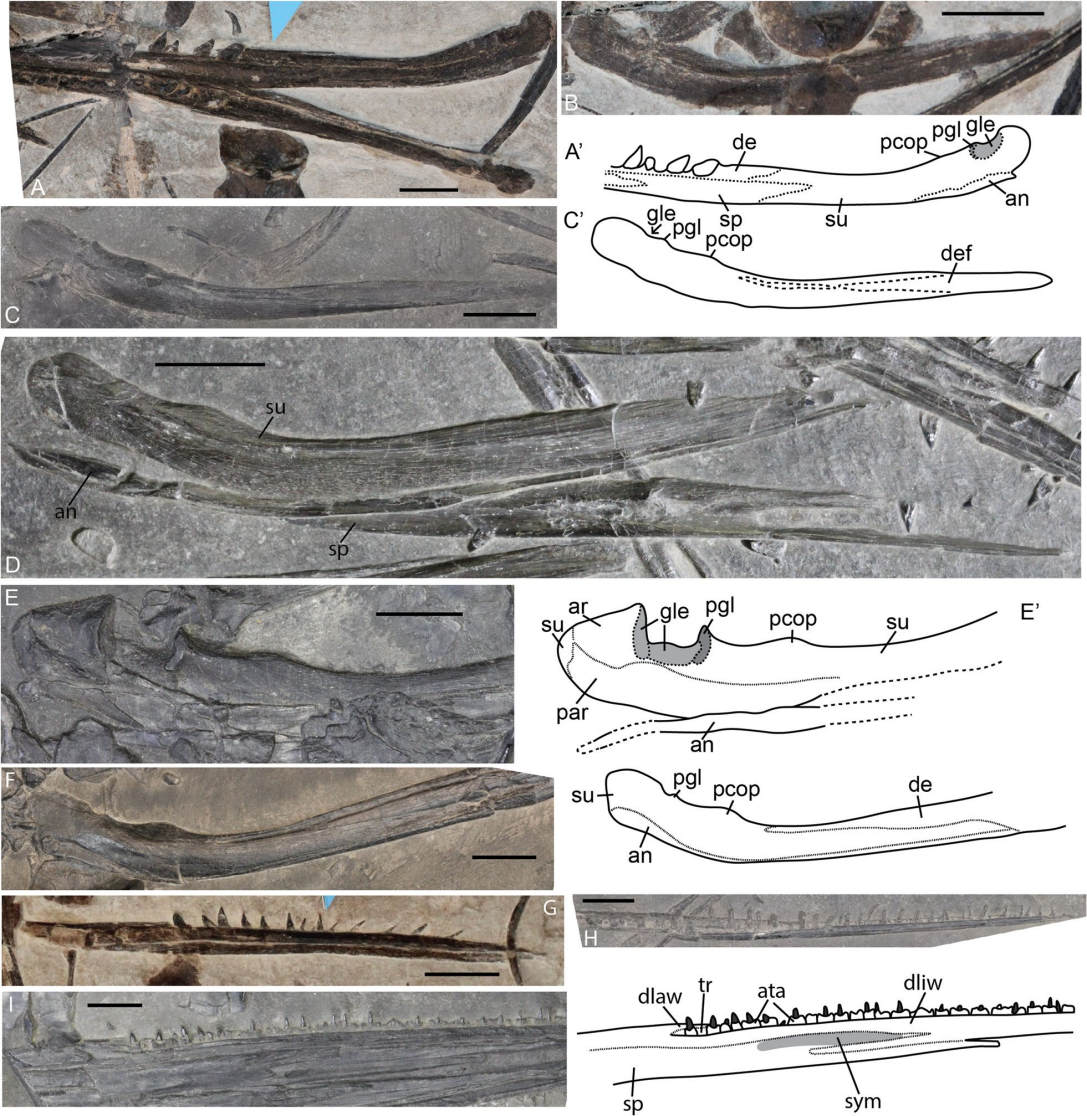
*Mixosaurus cornalianus*, lower jaw elements. Interpretative drawings are identified by an apostrophe beside their corresponding letter. **A** Articulated lower jaw of a fetus of PIMUZ T 4830 in medio-dorsal view (interpretative drawing only depicts right-side); **B** isolated surangular of fetus PIMUZ T 4830 in medial view; **C** isolated left surangular of a juvenile specimen PIMUZ T 2416 in lateral view; **D** disarticulated right lower jaw of a juvenile MSNM BES SC 1903 in lateral view; **E** posterior left lower jaw of adult specimen MSNM BES SC 1001 in medial view; **F** articulated posterior lower jaw of adult specimen PIMUZ T 2414 in lateral view; **G** isolated dentary of fetus of PIMUZ T 4830 in medial view; **H** isolated dentary of juvenile specimen PIMUZ T 2416 in medial view; **I** midsection left lower jaw of adult specimen MSNM BES SC 1001 in medial view (interpretative drawing emphasizes the splenial). *an* angular, *ar* articular, *ata* ankylosed tooth attachment, *de* dentary, *def* dentary facet, *dlaw* dentary labial wall, *dliw* dentary lingual wall, *gle* glenoid, *par* prearticular, *pcop* paracoronoid process, *pgl* preglenoid process, *sp* splenial, *su* surangular, *sym* symphyseal marks, *tr* tooth row. Scale bar equals 10 mm

The angular is a very slender element. It is equally deep along its length. This appears to be consistent over ontogeny (Fig. 14A, D–G). An angular of equal depth along its length is a trait seen in other more basal ichthyosauriforms; in Euichthyosauria the posterior portion is generally deeper than the anterior portion (Bindellini et al., 2021; Cuthbertson et al., 2013; Huang et al., 2019; Moon & Kirton, 2016).

The surangular of *Mixosaurus* is likewise very similar to that of most other ichthyosaurs. It is the deepest element forming most of the posterolateral portion of the lower jaw. The posterior portion has two dorsal projections. The more anterior is the paracoronoid process, which is a gradual bulge. More posteriorly and directly anterior to the glenoid contribution of the surangular lies the more dorsally tapering preglenoid process. Angle of preservation influences how large the processes appear relative to the surangular, whereby medially the processes appear larger than they do laterally in sexually mature specimens (Fig. 14B–F). In lateral view, the paracoronoid process becomes relatively larger over postnatal ontogeny (Fig. 14B–F).

The splenial forms most of the medial wall of the mandible. It is an anterior–posteriorly elongated element. Anteriorly it consists of two distinct prongs, the dorsal one shows distinct striations indicating its contribution to the symphysis in all postnatal specimens studied. No clear ontogenetic morphological variation was observed. The mandibular symphysis is already well established perinatally (Fig. 14A).

The dentary forms the anterior and only tooth-bearing portion of the lower jaw. Unlike the maxilla, the dentary does not contain distinct tooth sockets. However, teeth do seem to have some form of ankylosis to the tooth row (Fig. 14H–I).

A distinct ossified coronoid separate from the surangular was not observed in any of the specimens. This contrasts with previous interpretations (Maisch & Matzke, 2000).

## Discussion

### Ontogenetically variable characters separating juvenile and adult specimens

Skull roof: the midline contact of the skull roof in juvenile specimens is only in contact and not fused, whereas it is fused in adult specimens. This can be seen by the universal disarticulation of the two skull roof halves in small postnatal specimens, whereas this is not seen in adults. Basioccipital: the ventral anterior margin of the extracondylar area of the basioccipital is invaginated in juvenile, whereas it forms a ventral depression in adults. Stapes: the medial head of the stapes lacks a dorsally projecting process in some juvenile specimens, but a larger sample size is required to consider this trait as conclusive. Exoccipital: the two portions of the basioccipital facet of the exoccipital are in contact below the hypoglossal foramen, giving the foramen an oval outline and the suture is often visible in immature specimens, the suture is obliterated and the hypoglossal foramen becomes rounder in outline around sexual maturity. Surangular: the paracoronoid process is more prominent in adults than in juvenile specimens.

### New interpretation of the *Mixosaurus* opisthotic and prootic

Our interpretations of the opisthotic and prootic are markedly different from those proposed by Maisch et al. (2006), however, the element we identify as an opisthotic in *Mixosaurus cornalianus* is similar to the opisthotic in the mixosaurid *Phalarodon fraasi* (Økland et al., 2018). We identify the element considered to be the otic capsule by Maisch et al. (2006) in PIMUZ T 4848 (Fig. 3A, B) as the axial neural arch and spine, with diagenetic compression having fractured the neural arch pedicle from the spine. The structure is identical to the axial neural arch in other *Mixosaurus* specimens of the same ontogenetic stage (adult stage) (Fig. 3A, B). Similar atlas–axis morphology is also observed in *Phalarodon fraasi* (Økland et al., 2018)*.*

### Early ontogenetic morphology of *Mixosaurus* and its phylogenetic position

The general degree of ossification of the cranial fetal material of PIMUZ T 4830 is similar to fetal stage 4 in *Stenopterygius* (Miedema & Maxwell, 2022). The chondrocranium, especially the basioccipital, lags behind the dermatocranial elements in terms of the degree of ossification. The dermatocranium, including skull roof and palatal elements, is well ossified.

The ossification centres still visible in the fetal stage include those of the parietal, frontal, lacrimal, and vomer. The ossification centres of the parietal and frontal are located over the optic lobe and olfactory lobe indentations, respectively (Fig. 12A), the lacrimal ossification centre is located over the lacrimal medial depression (Fig. 11A) and the centre of ossification of the vomer is at a posteroventral position, anterior to the presumed vomerine crest (Fig. 13E). These ossification centres are in the same positions as in *Stenopterygius*, with the exception of the vomer, showing that the two ichthyosaurs have broadly similar developmental patterns (Miedema & Maxwell, 2022) (Fig. 13F).

The vomer in *Mixosaurus* ossifies from a centre anterior to the vomerine crest, whereas in contrast, in *Stenopterygius* the ossification centre is situated ventral to the crest, i.e. the centre has shifted posteriorly in *Stenopterygius* (Fig. 13E, F). The vomerine crest has been interpreted as forming the medial wall of the choanal tube, and therefore it should be situated no more posteriorly than the posterior end of the internal narial opening. The position of the crest in both *Stenopterygius* and *Mixosaurus* is consistent with this interpretation; thus, we believe that the vomerine crest has been correctly identified. We hypothesize that the shift in the position of the ossification centre is related to the extent of the medial choanal process of the palatine, which is short in *Stenopterygius* (Miedema & Maxwell, 2022; Owen, 1881) but extends along half the length of the internal nares in *Mixosaurus cornalianus* (Maisch & Matzke, 1997a).

The ossification of the extracondylar area of the basioccipital of *Mixosaurus* is quite remarkable. In extant reptiles, the basioccipital ossifies from the basal plate. The notochord can be embedded in the basal plate (e.g., Sheil, 2005), can be exposed ventral to the basal plate, posterior to the basicranial fenestra, or can be exposed dorsal to the basal plate in the region of the basicranial fenestra, (e.g., Bellairs & Kamal, 1981). The basioccipital ossifies on the dorsal and ventral surfaces of the basal plate around the notochord. In the case of *Mixosaurus*, the notochord is likely exposed ventral to the basal plate, such that ossification proceeds around the notochord forming a furrow in the anterior basioccipital which gradually ossifies into a ventral depression postnatally. This depression divides the extracondylar area, forming basal tubera, as seen in other basal diapsids such as younginiforms (Gardner et al., 2010). Within Ichthyosauria, basal tubera have only been described in *Phantomosaurus* and *Shonisaurus*, and are absent in all Thunnosauria (Camp, 1980; Maisch & Matzke, 2000, 2006). The basioccipital is not (well) known in Nasorostra (= Omphalosauridae), Hupehsuchia, or non-ichthyosaurian ichthyopterygians so whether the structures observed in *Mixosaurus* are homologous with those of basal diapsids is unclear. While the midline furrow is present in late-stage fetuses of *Mixosaurus*, it is completely absent in stage 3 and 4 fetuses of *Stenopterygius* (Miedema & Maxwell, 2019), indicating that there is a change in development of the basicranium over ichthyosaurian phylogeny. This is plausibly the result of the notochord shifting from a position in which it pierces the basal plate in the fetal chondrocranium of *Mixosaurus*, to one in which it lies dorsal to the basal plate in *Stenopterygius*.

The parabasisphenoid of *Mixosaurus* likewise shows possible ancestral features. In adult specimens, there are processes projecting posterolaterally, which can be considered (remnants of the) basal tubera. Basal tubera are ubiquitously present in early diverging sauropsids (Ford & Benson, 2018; Gardner et al., 2010). Moreover, the overall more triangular outline and broad cultriform process is an ancestral character,in Euichthyosauria the basisphenoid is more quadrangular in outline and the cultriform process is narrow (Zverkov et al., 2021). Interestingly, the fetal specimen does show a slight constriction of the posterior parasphenoid as well as a more quadrangular basisphenoid, giving it an outline that is relatively more akin to the euichthyosaurian condition than the adult morphology. The parabasisphenoid outline is triangular in stage 1 *Stenopterygius*, but it also already shows a posteriorly narrow cultriform process of the parasphenoid (Miedema & Maxwell, 2022).

The exoccipitals in *Mixosaurus* are high and narrow relative to the state in *Stenopterygius*, with the initial ossification centre situated dorsal to the hypoglossal foramen and extending ventrally on either side and failing to merge ventrally prior to birth. This developmental pattern requires further investigation, but might indicate a gradual dorsal displacement of the foramen over ichthyosaurian evolution, from a primitive position at the basioccipital–exoccipital suture. A large foramen situated within the body of the exoccipital is already present in both non-ichthyosaurian ichthyopterygians (*Grippia*: Maxwell & Kear, 2013) and hupehsuchians (Carroll & Dong, 1991), however placement of the hypoglossal foramen on the basioccipital–exoccipital suture is observed in basal sauropterygians (placodonts: (Neenan & Scheyer, 2012). A large foramen on the basioccipital–exoccipital suture is also present in thalattosaurs, but was identified as the jugular foramen (Liu & Rieppel, 2005). However, *Nothosaurus marchicus* (eosauropterygia) and *Askeptosaurus italicus* do have hypoglossal foramina completely enclosed by the exoccipital (Müller, 2005; Voeten et al., 2018). The earliest known prenatal exoccipital of *Stenopterygius* is stage 3. It shows the foramen completely enclosed in the exoccipital ossification, but ventrally placed (Miedema & Maxwell, 2019, Fig.  4I). Although no ventral suture can be seen it is possible that the enclosure of the hypoglossal foramen has the same developmental trajectory, but beginning much earlier in development. The ontogenetic trajectory of the exoccipital in *Mixosaurus* is unusual, but its significance remains unclear: while potentially indicating a conserved primitive ontogenetic trajectory, it cannot be ruled out that the unique ossification pattern is something specific to mixosaurids. Other braincase and all splanchnocranial elements resemble standard euichthyosaurian morphology.

The ontogenetic change in postorbital morphology is also reminiscent of the hypothetical ancestral state. In early-diverging diapsids, the postorbital is triradiate, contacting the squamosal posteriorly, the frontal and/or postfrontal anteriorly and the jugal ventrally to form the division between the upper and lower temporal fenestrae (UTF and LTF) (e.g., Osborn, 1903). This state is seen in many early diapsids and still persists in some lepidosaurs (Currie, 1981; Evans, 2008; Gow, 1975; Martínez et al., 2021; Miedema et al., 2020; Sobral et al., 2015). Hupehsuchians and the earliest ichthyosauriforms still show a clearly triradiate postorbital in adults, with the postorbital forming the border between the UTF and open LTF but not participating in the latter (Huang et al., 2019; Jiang et al., 2016; Motani et al., 2015; Wu et al., 2016; Zhou et al., 2017). Fetal material of *Mixosaurus* shows a triradiate postorbital morphology, although there is no posterior process but rather two dorsal processes (Fig. 10E) that form a single sheet postnatally with no recognizable processes (Fig. 10F). The developmental condition in *Mixosaurus* shows that it has not lost the original ossification template including dorsal processes, which are then also likely still present in prenatal stages of other early ichthyosauriforms. *Stenopterygius* has lost the triradiate postorbital morphology even in the earliest documented developmental stages (Miedema & Maxwell, 2022), which again attests to a developmental shift over evolutionary history between Triassic ichthyosauriforms and parvipelvians.

### Paleobiological implications of perinatal morphology

Similar to *Stenopterygius*, the degree of ossification is high in the cranium of perinatal *Mixosaurus* specimens (Miedema & Maxwell, 2022). Likewise, the appendicular and postcranial axial skeleton is well-ossified (Brinkmann, 1996). Moreover, the mandibular symphysis is well established perinatally and the dentition is similar to that of adults, indicating that the jaws were fully functional. Neonates were therefore likely relatively precocial. Therefore, as in *Stenopterygius*, post-parturitional parental care is not impossible, but is not supported by osteological evidence.

Also similar to perinatal *Stenopterygius* is the weak contact between the two halves of the skull roof (Miedema & Maxwell, 2022). The contacts between parietal, frontal and nasal are well-established perinatally, but the antimeric contacts are absent at this stage (Fig. 12A). The contacts are likewise not well established in the “juvenile” stages as many small postnatal specimens are preserved with disarticulated skull roof halves, even though the non-midline sutures are fully closed. This further corroborates that delayed midline fusion in the skull roof of ichthyosaurs functions as a fontanelle to avoid damage during birth (Miedema & Maxwell, 2022).

## Conclusions

The ontogenetic trajectory of the cranium of *Mixosaurus* is quite interesting as it shows hints of the developmental pathways that changed in ichthyosaurs from their sauropsid ancestors. The basioccipital extracondylar area is split prenatally and early postnatally, in contrast to the development in *Stenopterygius*. We deem it likely that the developmental pathway that led to the formation of basal tubera in early diverging diapsids is still present in *Mixosaurus*, but absent in *Stenopterygius*. Remnants of basal tubera are also still visible in the adult morphology of the parabasisphenoid. Likewise, we observed a triradiate postorbital in the prenatal stage, but not postnatally. Triradiate morphology is still present in some early diverging ichthyosauromorphs and most early diapsids, whereas a more lunate shape is common in Euichthyosauria. Unfortunately, due to sample size and preservation we were only able to identify a few reliable postnatally ontogenetically varying characters in the basioccipital (closure of the extracondylar area midline), exoccipital (closure of the suture between the feet), stapes (prominence of the dorsal portion of the medial head), surangular (prominence of the paracoronoid process) and skull roof (midline suture establishment). The prenatal and postnatal open midline suture is also seen in *Stenopterygius* and is further evidence for this area serving as a fontanelle in ichthyosaurs.

### Supplementary Information

**Additional file 1.** Metric data for *Mixosaurus cornalianus* specimens studied.

## Data Availability

All specimens are available for study at their respective museums. Please contact Christian Klug (PIMUZ), Ingmar Werneburg (GPIT), Cristiano Dal Sasso (MSNM) and Erin Maxwell (SMNS) for respective collection access.

## Abbreviations

GPIT: Geologisches und Paläontologisches Institut Tübingen, Tübingen, Germany
MSNM: Museo di Storia Naturale di Milano, Milan, Italy (with BES SC as the acronym for Besano, Sasso Caldo site)
PIMUZ: Paläontologisches Institut und Museum der Universität Zürich, Zürich, Switzerland
SMNS: Staatliches museum für Naturkunde Stuttgart, Stuttgart, Germany
